# Oncogene-dependent function of BRG1 in hepatocarcinogenesis

**DOI:** 10.1038/s41419-020-2289-3

**Published:** 2020-02-04

**Authors:** Pan Wang, Xinhua Song, Dan Cao, Kairong Cui, Jingxiao Wang, Kirsten Utpatel, Runze Shang, Haichuan Wang, Li Che, Matthias Evert, Keji Zhao, Diego F. Calvisi, Xin Chen

**Affiliations:** 1 Department of Bioengineering and Therapeutic Sciences, University of California, San Francisco, CA USA; 2 Department of Medical Oncology, Cancer Center, State Key Laboratory of Biotherapy, West China Hospital, Sichuan University, Chengdu, Sichuan China; 3 Systems Biology Center, NHLBI, NIH, 9000 Rockville Pike, Bethesda, MD 20892 USA; 4 Institute of Pathology, University of Regensburg, Regensburg, Germany; 5 Department of Hepatobiliary Surgery, Xijing Hospital, Fourth Military Medical University (Air Force Medical University), Xi’an, China; 6 Department of Hepatobiliary Surgery, Xijing Hospital, Air Force Military Medical University, Xi’an, China; 7 Department of Medical, Surgical, and Experimental Sciences, University of Sassari, Sassari, Italy

**Keywords:** Cancer genomics, Cancer models

## Abstract

Hepatocellular carcinoma (HCC) is the major type of primary liver cancer. Genomic studies have revealed that HCC is a heterogeneous disease with multiple subtypes. BRG1, encoded by the *SMARCA4* gene, is a key component of SWI/SNF chromatin-remodeling complexes. Based on TCGA studies, somatic mutations of *SMARCA4* occur in ~3% of human HCC samples. Additional studies suggest that BRG1 is overexpressed in human HCC specimens and may promote HCC growth and invasion. However, the precise functional roles of BRG1 in HCC remain poorly delineated. Here, we analyzed BRG1 in human HCC samples as well as in mouse models. We found that BRG1 is overexpressed in most of human HCC samples, especially in those associated with poorer prognosis. BRG1 expression levels positively correlate with cell cycle and negatively with metabolic pathways in the Cancer Genome Atlas (TCGA) human HCC data set. In a murine HCC model induced by c-MYC overexpression, ablation of the *Brg1* gene completely repressed HCC formation. In striking contrast, however, we discovered that concomitant deletion of *Brg1* and overexpression of c-Met or mutant NRas (NRAS^V12^) triggered HCC formation in mice. Altogether, the present data indicate that BRG1 possesses both oncogenic and tumor-suppressing roles depending on the oncogenic stimuli during hepatocarcinogenesis.

## Introduction

Primary liver cancer is the sixth most common tumor in the world^1^. Hepatocellular carcinoma (HCC) is the predominant subtype of liver cancer. Owing to the lack of specific symptoms, most HCCs are diagnosed at advanced stage. Therapeutic approaches for advanced HCC are limited. Indeed, these HCC patients are eligible to treatment with Sorafenib and Regorafenib multi-kinase inhibitors, but only limited benefits are observed^2^. Recently, immune checkpoint inhibitors have also been approved as second line treatment with marked responses, but only in ~20% of patients^3^. Thus, it is imperative to elucidate the molecular mechanisms underlying hepatocarcinogenesis in order to develop innovative and more effective therapies against HCC.

ATP-dependent chromatin remodeling is involved in controlling chromatin structure that in turn regulates many physiological and pathological processes^4^. Switching/sucrose non-fermentable (SWI/SNF) complexes are members of the family of ATP-dependent chromatin-remodeling complexes, which consists of ~15 subunits^5^. Mammalian SWI/SNF complexes family further divided into two major complexes, including the BRG1-associated factor (BAF) complex and the poly-bromo BRG1-associated factor (PBAF) complexes^6^. All complexes contain one of two mutually exclusive catalytic ATPase subunits, either BRG1 (encoded by *SMARCA4*) or BRM (encoded by *SMARCA2*)^7^. The function of SWI/SNF is to regulate gene transcription by rearranging nucleosome positions and histone–DNA interactions, thus facilitating the transcriptional activation or repression of target genes^4^. The complexes control a wide variety of physiological and cellular processes, including tissue specificity, inflammatory processes, immunological responses, and early embryonic development^8^. Recent exome sequencing studies have revealed that SWI/SNF subunits of chromatin-remodeling complexes are mutated in over 20% of human tumors^7^. The key role of SWI/SNF in HCC has been highlighted by the fact that the SWI/SNF complex subunits, *ARID1A* and *ARID2*, are mutated in 7% and 5% of human HCC samples, respectively^9^, thus representing some of the most mutated genes in HCC.

BRG1, the core subunit of the SWI/SNF complex, is essential for DNA repair, differentiation, and organ development^10^. However, the role of mutated BRG1 in tumorigenesis remains largely controversial. Although some human tumors show overexpression of BRG1, other display instead a suppression of BRG1 expression^11–14^. In addition, BRG1 can interact with both proliferation promoting and inhibiting genes^15,16^. Altogether, these data envisage the possibility that BRG1 may function either as an oncogene or tumor suppressor gene in a context-dependent manner. The contradictory function of BRG1 has been also observed in human HCC. For instance, it has been shown that the SNU398 HCC cell line harbors a homozygous deletion of the *SMARCA4* gene. In addition, copy number loss at *SMARCA4* locus was detected in 14% of primary HCC tumors^11^. In striking contrast, a positive correlation between increased BRG1 expression and HCC aggressiveness was described^17^. Furthermore, Benedikt et al.^18^ showed that BRG1 promotes hepatocarcinogenesis by regulating proliferation and invasiveness.

In this manuscript, we systematically analyzed BRG1 expression as well as *SMARCA4* mutation status in human HCC samples. Using conditional *Brg1* KO mice and oncogene-driven HCC murine models, we investigated the functional role(s) of Brg1 in hepatocarcinogenesis. Our data support the hypothesis that BRG1 functions predominantly as an oncogene in HCC. However, BRG1 possesses also a tumor suppressive role in a small percentage of human HCCs.

## Results

### BRG1 expression and *SMARCA4* mutation status in human HCC samples

**Fig. 1:**
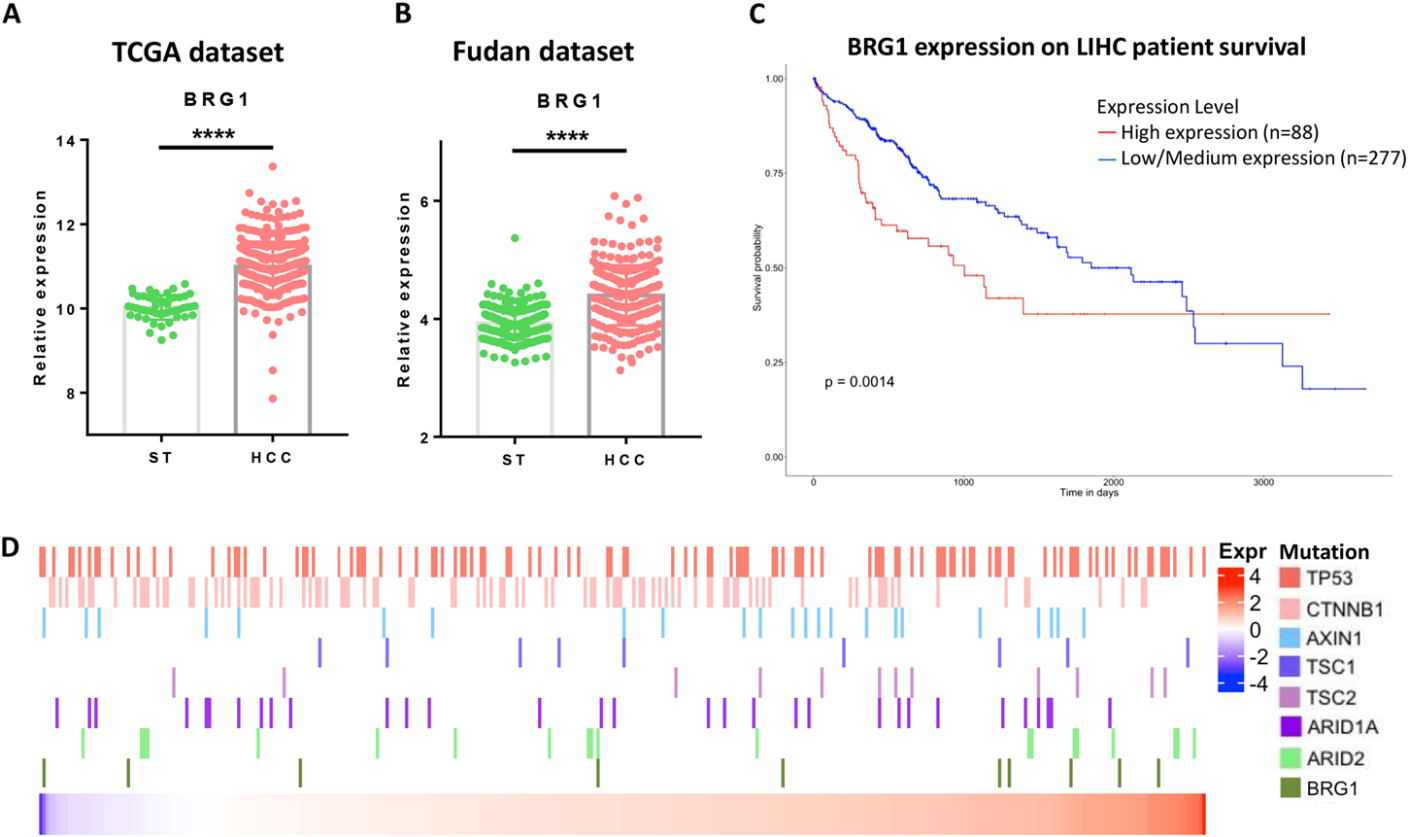
BRG1 expression, mutation, and survival analysis in human data sets. **a** Scatter-bar plot of BRG1 mRNA expression in TCGA LIHC data set. **b** Scatter-bar plot of BRG1 mRNA expression in Fudan data set. **c** Kaplan–Meier survival plot from UALCAN using TCGA LIHC data set. **d** Heatmap of BRG1 mRNA expression in TCGA LIHC data set with multiple mutation status of well-known oncogenes in HCC. ST surrounding tissue, HCC hepatocellular carcinoma; ****p < 0.0001.

To study BRG1 in human HCCs, we first analyzed BRG1 expression levels using TCGA human HCC data set. We discovered that BRG1 expression levels are upregulated in most human HCC samples when compared with non-tumor liver tissues (Fig. 1a), although ~ 3% of HCCs have lower BRG1 expression. This result was independently validated via the Fudan HCC data set (Fig. 1b). Consistent with a previous report^19^, high expression of BRG1 is associated with poor HCC patient survival (Fig. 1c).

To further validate these results, we evaluated *BRG1* mRNA levels in our collection of human normal livers, HCCs, and corresponding non-tumorous surrounding livers (*n* = 60; Supplementary Fig. 1A). We found that mRNA expression of *BRG1* was significantly higher in HCC when compared with non-tumorous surrounding livers and normal livers (Supplementary Fig. 1A). Furthermore, the most pronounced upregulation of *BRG1* was detected in human HCC with poorer prognosis (HCCP; Supplementary Fig. 1B). No significant association between the mRNA levels of *BRG1* and clinicopathologic features of the patients, such as gender, etiology, presence of cirrhosis, alpha-fetoprotein levels, and tumor size was found (data not shown). We also performed immunostaining of BRG1 in paired human HCC and surrounding non-tumor liver tissues. Again, we found that BRG1 is expressed at higher levels in most human HCC samples (Supplementary Fig. 2A, B). However, a small percentage of human HCCs show very low BRG1 protein expression (Supplementary Fig. 2C, E). In most of the non-tumor liver tissues, there is minimum expression of BRG1 in hepatocytes, whereas strong BRG1 expression could be detected in bile duct cells, lymphocytes, and macrophages (Supplementary Fig. 2D).

Next, we explored *SMARCA4* mutation status in human HCC samples. Among the 360 HCCs in the TCGA database, 10 HCCs harbored a *SMARCA4* mutation (Supplementary Fig. 3A). As *SMARCA4* mutations are rather rare, we expand the search into COSMIC database^20^ (Supplementary Fig. 3B). In total, *SMARCA4* mutation rate was found to be ~2.4% in human HCCs. It is worth to note that *SMARCA4* mutations are generally point mutations in HCCs, and these mutations scatter throughout the BRG1 protein sequence, with no clear hot spots (Supplementary Fig. 3B).

We then analyzed *SMARCA4* mutation and BRG1 expression levels in relationship to other common mutations, including mutations in *TP53*, *CTNNB1*, *AXIN1*, *TSC1/2,* and *ARID1A/2* in human HCC samples (Fig. 1d). We did not observe any statistically significant correlation between *SMARCA4* mutations or BRG1 expression levels with these common genetic events in human HCCs.

**Fig. 2:**
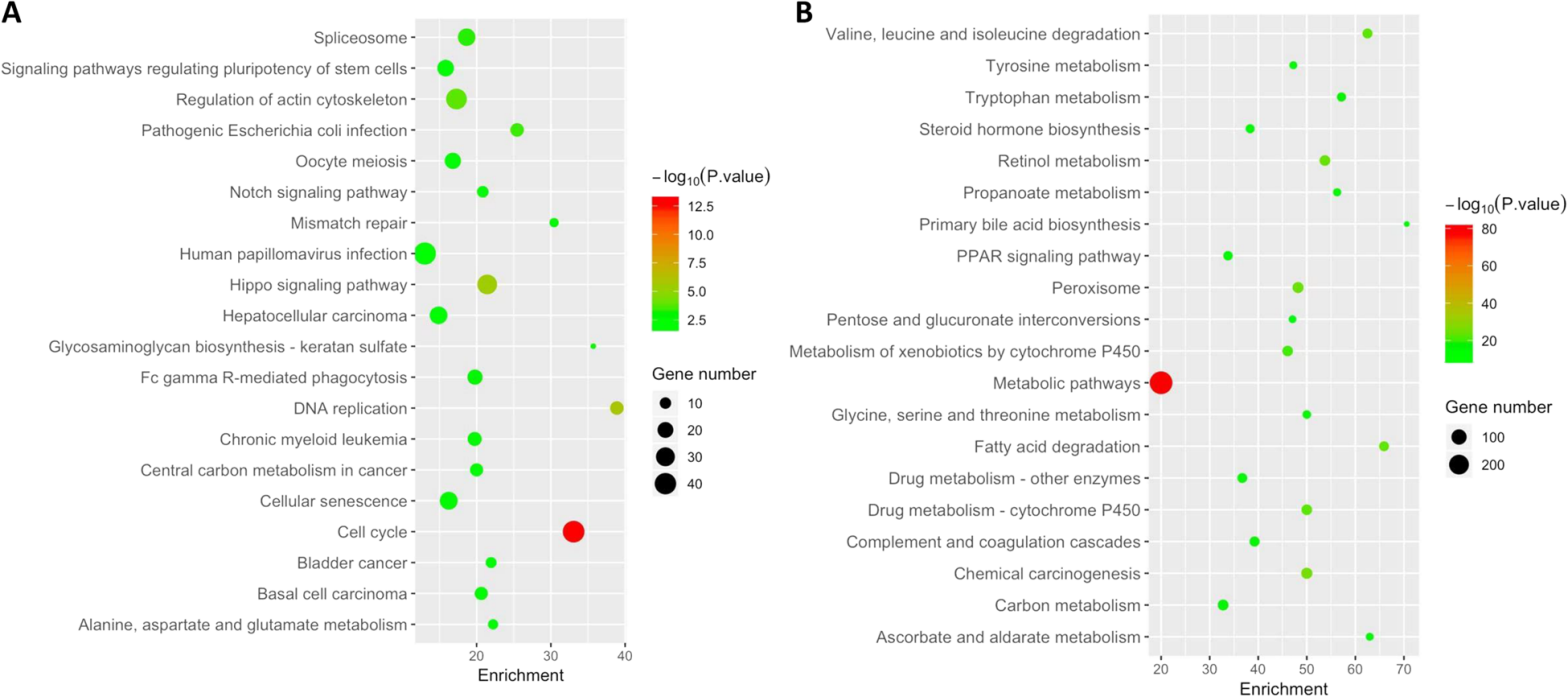
KEGG pathway analysis of BRG1 co-expressed genes. **a** Positively co-expressed genes. **b** Negatively co-expressed genes. Enrichment number means the percentage of BRG1 co-expressed genes in the pathway.

Finally, we analyzed the genes whose expression levels correlate with BRG1 mRNA expression in human HCCs using the TCGA data set^9^. We identified 1915 genes whose expression levels positively correlate with BRG1 mRNA level, and 1067 genes negatively correlate with BRG1 mRNA level (Supplementary Tables 1, 2). Among the positively co-expressed genes, BRG1 expression had a strong correlation with CDK4, CCNB1, and CCNE1 (Supplementary Fig. 4). KEGG pathway analysis revealed that BRG1 positively co-expressed genes were significantly enriched in cell cycle pathway and DNA replication and HCC (Fig. 2a), which further implicates the role of BRG1 in promoting HCC proliferation. Interestingly, BRG1 negatively co-expressed genes were highly enriched in metabolic pathways, including vital hepatocyte functions, such as primary bile acid biosynthesis, drug metabolism, steroid hormone biosynthesis, etc. The result indicates that high BRG1 expression correlates with poorly differentiated status in human HCC (Fig. 2b). Similar results were obtained in GO analysis (Supplementary Fig. 5).

In summary, our findings indicate that BRG1 expression is upregulated in most human HCC samples, and high expression of BRG1 correlates with poor prognosis. At the molecular level, BRG1-elevated levels are positively associated with increased HCC cell proliferation and decreased differentiation status. However, mutations in *SMARCA4* or BRG1 low expression can be found in a small percentage of human HCCs. Thus, based on expression levels, BRG1 may predominantly function as an oncogene along hepatocarcinogenesis, but can also act as a tumor suppressor in a small percentage of human HCC.

### Loss of Brg1 does not affect liver homeostasis

**Fig. 3:**
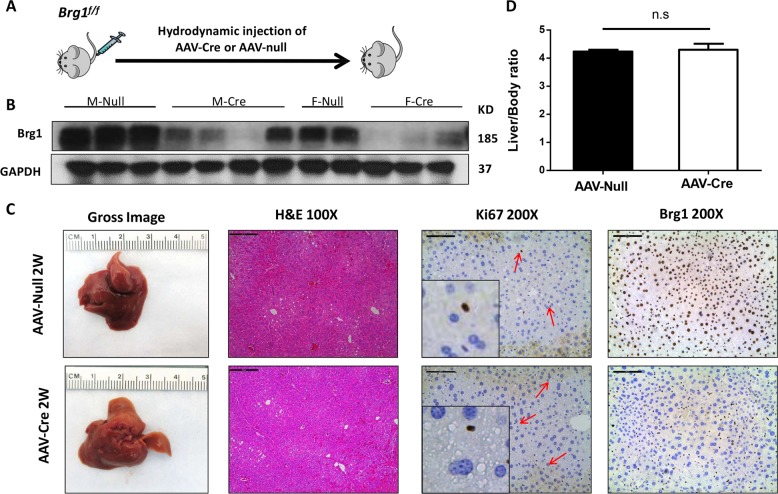
Ablation of Brg1 in mouse hepatocytes does not affect liver homeostasis in adult mice. **a** Study design. **b** Protein expression of Brg1 in the liver of *Brg1*^*flf*^ AAV-Cre and of *Brg1*^*flf*^ AAV-null mice analyzed by western blot analysis. **c** Gross image, H&E staining, Ki67, and Brg1 staining of *Brg1*^*flf*^ AAV-Cre and of *Brg1*^*flf*^ AAV-null mice. **d** Liver body weight ratio of *Brg1*^*flf*^ AAV-Cre (*n* = 6) and *Brg1*^*flf*^ AAV-null mice (*n* = 7).

To investigate the functional role(s) of Brg1 in hepatocarcinogenesis, we applied genetic approaches using the *Brg1*^*f/f*^ mice. First, we investigated whether ablation of *Brg1* affects liver homeostasis. For this purpose, we injected *Brg1*^*f/f*^ mice with adeno-associated virus encoding null (AAV-null) or Cre (AAV-Cre) plasmid under liver specific thyroxine-binding globulin (TBG) promoter (Fig. 3a). We found that AAV-Cre infection effectively deleted Brg1 in mouse hepatocytes (Fig. 3b, c) in both male and female mice. The residual Brg1 expression detected in AAV-Cre mice by western blotting reflected Brg1 expression in non-parenchymal cells in the liver, which could be clearly visualized by immunostaining (Fig. 3c). Consistent with a previous report^21^, we found that loss of *Brg1* did not affect liver homeostasis: indeed, liver/body weight ratio (Fig. 3d), liver histology as well as hepatocyte proliferation (Fig. 3c) were equivalent in AAV-null and AAV-Cre mice.

To identify the genes regulated by Brg1, we compared gene expression patterns of liver tissues from AAV-Null and AAV-Cre mice using RNASeq. We discovered that 68 genes were upregulated, and 79 genes downregulated in Brg1(−) liver tissues when compared with Brg1(+) liver tissues (Supplementary Figs. 6, 7; and Supplementary Table 3). Several selected genes were further validated using qRT-PCR, supporting the reliability of RNASeq results (Supplementary Fig. 8). Despite the fact that Brg1 is a core component of the SWI/SNF complex, loss of *Brg1* had a relatively limited impact on global gene expression patterns. These findings suggest that additional molecules in the complex may compensate for the loss of Brg1 to preserve liver homeostasis.

### Loss of Brg1 inhibited c-MYC driven hepatic carcinogenesis

Amplification of c-MYC is one of the most frequent genetic events along HCC development and progression^22^. In mice, hydrodynamic transfection of c-MYC rapidly induces the formation of poorly differentiated HCC, and mice have to be euthanized owing to high liver tumor burden by ~6 weeks post hydrodynamic injection^23^, supporting the oncogenic role of c-MYC in the liver.

We found that nuclear Brg1 staining could be readily detected in c-MYC mouse HCC tissues (Supplementary Fig. 9). We have previously performed global gene expression analysis of c-MYC mouse HCC and normal mouse liver tissues using microarrays^23^. We investigated whether BRG1-correlated genes are differentially expressed in c-MYC mouse HCCs. Specifically, 1495 BRG1 positively correlated genes and 799 BRG1 negatively correlated genes were identified in the mouse data set. Heatmap analysis revealed that most of the BRG1 positively correlated genes were upregulated in c-MYC HCC samples, whereas most of the BRG1 negatively correlated genes were downregulated in c-MYC HCC (Supplementary Fig. 10A, B). Indeed, we found that 11.6% of total genes were upregulated in c-MYC HCC, 26.3% of the BRG1 positively correlated genes were upregulated, and 3.6% of BRG1 negatively correlated genes were upregulated. Similarly, 9.5% of total genes were downregulated in c-MYC HCC. 2.9% of the BRG1 positively correlated genes were downregulated, and 47.9% of BRG1 negatively correlated genes were downregulated (Supplementary Fig. 10C). Altogether, our study demonstrates the Brg1 is upregulated in c-MYC mouse HCC, leading to the activation of Brg1-correlated gene expression signature.

**Fig. 4:**
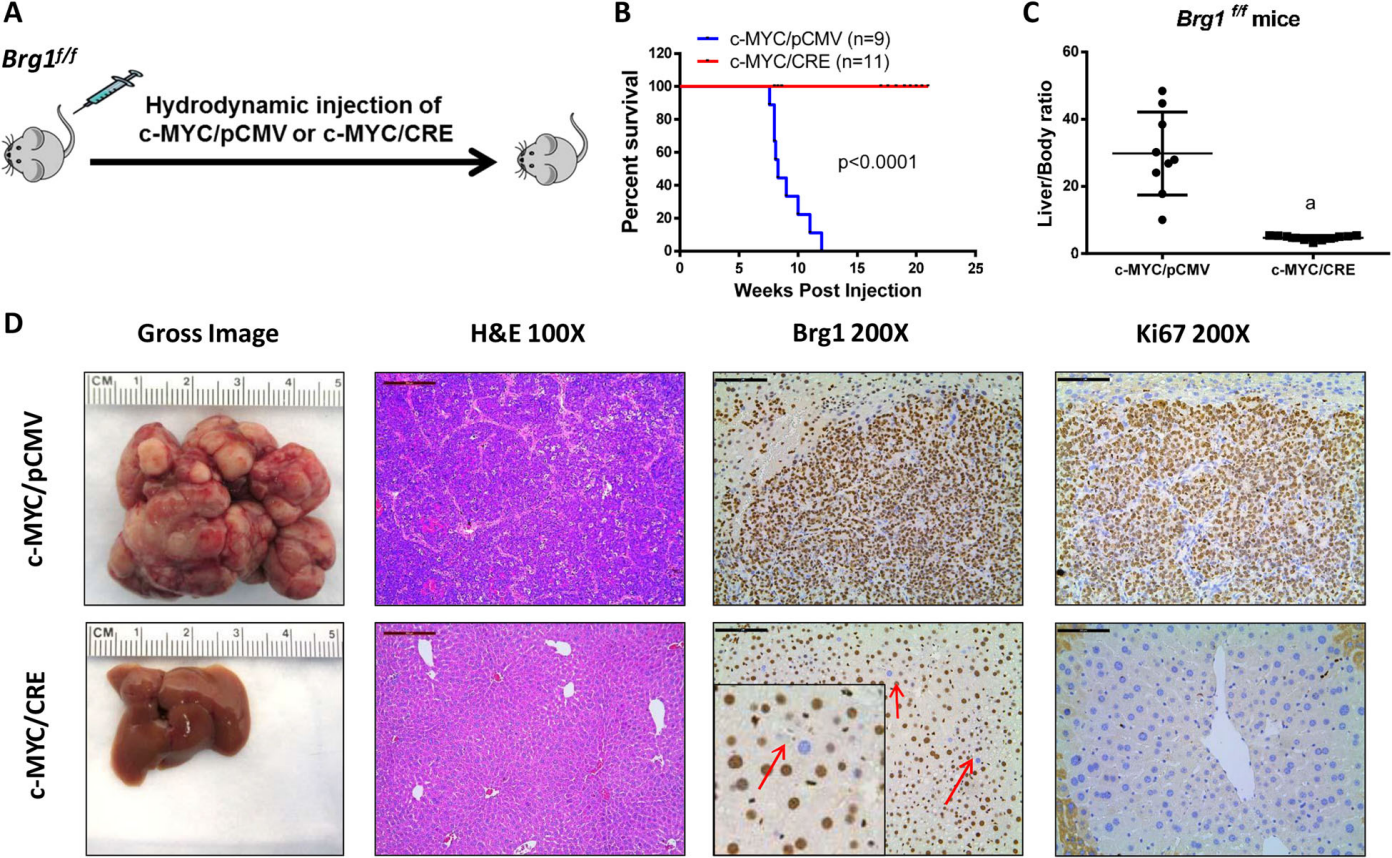
Brg1 inactivation prevents hepatocellular carcinoma (HCC) formation in c-MYC mice. **a** Study design. **b** Survival analysis of Brg1^*f/f*^ mice bearing c-MYC/pCMV and c-MYC /CRE tumors using the Kaplan–Meier survival method. **c** Liver body weight ratio of *Brg1*^*f/f*^ c-MYC/pCMV (*n* = 9) and c-MYC /CRE mice (*n* = 11). **d** Gross image, H&E staining, Brg1, and Ki67 staining of *Brg1*^*f/f*^ c-MYC/pCMV and c-MYC /CRE mice.

To investigate the functional contribution of Brg1 in c-MYC driven HCC, we hydrodynamically injected c-MYC with CRE plasmids into *Brg1*^*f/f*^ mice (c-MYC/CRE) (Fig. 4a). This approach allowed the overexpression of c-MYC oncogene while simultaneously deleting *Brg1* in the same set of mouse hepatocytes. As control, additional *Brg1*^*f/f*^ mice were injected with c-MYC and pCMV empty vector plasmid (c-MYC/pCMV) (Fig. 4a). All c-MYC/pCMV injected *Brg1*^*f/f*^ mice developed high liver tumor burden and became moribund between 7.6 and 12 weeks post injection. In striking contrast, all c-MYC/CRE injected *Brg1*^*f/f*^ mice appeared to be healthy with no signs of tumor development (Fig. 4b). Consistently, c-MYC/pCMV mice had high liver/body weight ratio, whereas c-MYC/CRE mice demonstrated similar ratio to that seen in normal mice (Fig. 4c). Grossly, numerous tumor nodules could be found in c-MYC/pCMV liver, but not in c-MYC/CRE livers (Fig. 4d). Histological evaluation revealed the poorly differentiated and highly proliferative (as shown by immunostaining of Ki67) HCC in c-MYC/pCMV mice. All tumor cells were Brg1(+). In contrast, histologically normal liver was detected in c-MYC/CRE mice with few Ki67(+) cells. It is important to clarify that we applied hydrodynamic injection in combination with sleeping beauty-mediated somatic integration to stably express genes into the mouse hepatocytes. This approach could stably transfect ~1–5% of mouse hepatocytes^24^. When we transfected c-MYC/CRE into *Brg1*^*f/f*^ mice, we overexpressed c-MYC while deleting *Brg1* in a small percentage of hepatocytes. Indeed, using immunostaining, sporadic Brg1(−) hepatocytes could be readily detected in c-MYC/CRE injected *Brg1*^*f/f*^ mouse liver tissues (Fig. 4d), supporting the successfulness of the expression of the injected plasmids. Nonetheless, these transfected hepatocytes were unable to progress into tumor cells.

Altogether, these findings demonstrate that Brg1 is indispensable for c-MYC induced HCC formation in vivo.

### Loss of Brg1 cooperates with c-MET or RAS^V12^ to promote HCC development in mice

Loss of BRG1 expression and *SMARCA4* loss-of-function mutations were identified in a small subset of human HCC samples, suggesting that BRG1 may also function as a tumor suppressor. To test this hypothesis, we overexpressed CRE in *Brg1*^*f/f*^ mice, with consequent deletion of Brg1 in a small subset of mouse hepatocytes. We found that sporadic deletion of *Brg1* alone is unable to promote liver tumor formation (Supplementary Fig. 11). This observation is consistent with our studies expressing AAV-CRE into *Brg1*^*f/f*^ mice, resulting in the deletion of Brg1 in all hepatocytes (Fig. 3). Overall, these results suggest that loss of Brg1 per se is not oncogenic.

**Fig. 5:**
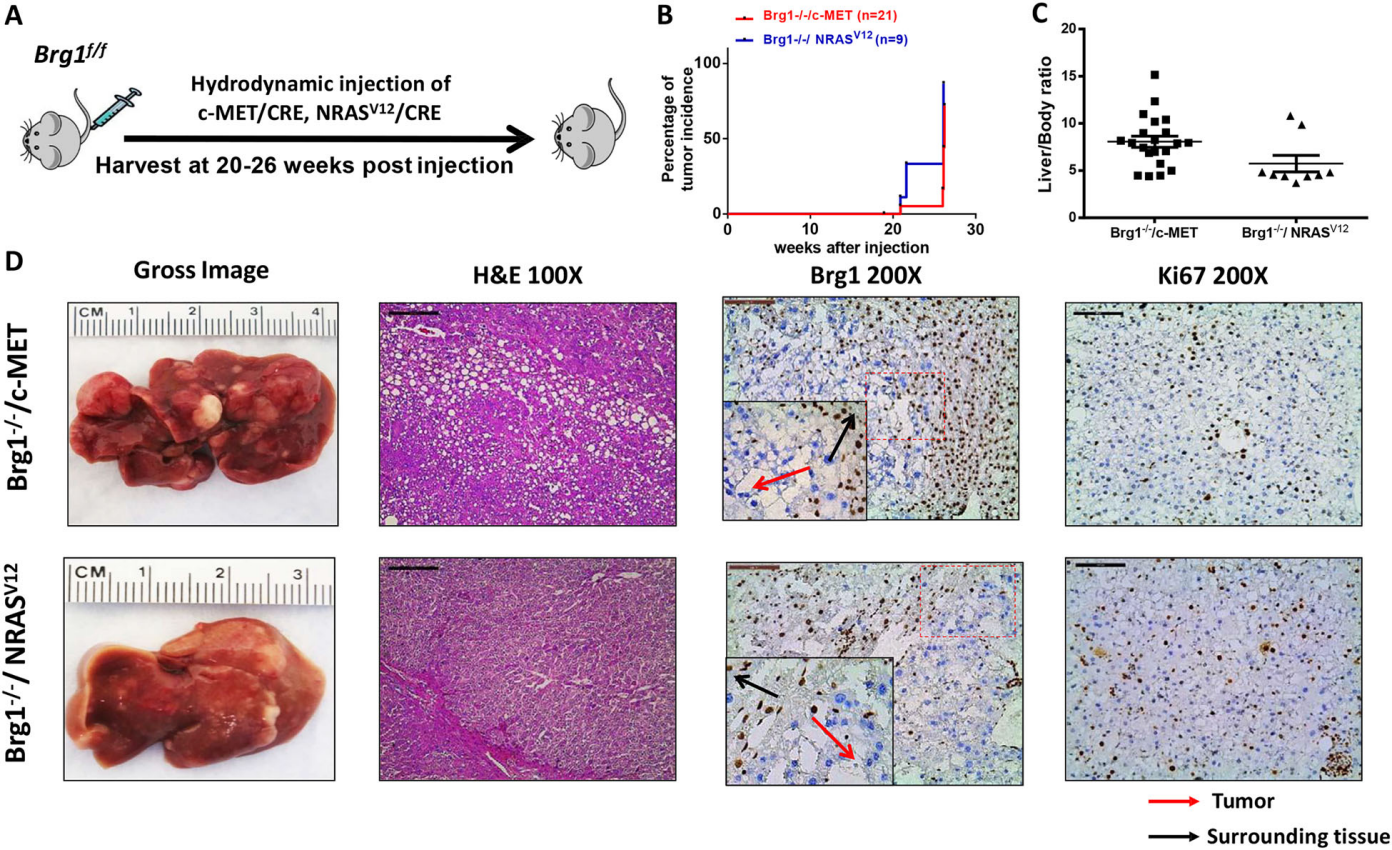
*Brg1* deletion cooperates with c-MET and NRAS^V12^ to induce liver tumor formation in mice. **a** Study design. **b** Survival analysis of *Brg1*^*f/f*^ mice injected CRE plasmid and c-MET (Brg1^−/−^/c-MET) or NRAS^V12^ (Brg1^−/−^/ NRAS^V12^). **c** Liver body weight ratio of Brg1^−/−^/c-MET (*n* = 21) and Brg1^−/−^/ NRAS^V12^mice (*n* = 9). **d** Gross image, H&E staining, Brg1, and Ki67 staining of Brg1^−/−^/c-MET and Brg1^−/−^/ NRAS^V12^mice.

Previous data indicate the ubiquitous activation of the RAS/MAPK signaling in HCC^25^, supporting a pivotal role of this signaling cascade along hepatocarcinogenesis. In the mouse liver, overexpression of either c-MET or an activated mutant form of NRAS (NRAS^V12^) is able to activate the RAS/MAPK signaling, although none of these genes alone is capable of inducing HCC formation in vivo^26^. Thus, we hypothesized that loss of *Brg1* may cooperate with c-MET or NRAS^V12^ to induce liver tumor development in mice. To validate our hypothesis, we hydrodynamically injected *Brg1*^*f/f*^ mice with CRE plasmid and c-MET (Brg1^−/−^/c-MET) or NRAS^V12^ (Brg1^−/−^/ NRAS^V12^) (Fig. 5a). Strikingly, liver tumors developed in Brg1^−/−^/c-MET and Brg1^−/−^/ NRAS^V12^ mice by 20–30 weeks post injection (Fig. 5b, c). Grossly, numerous tumor nodules were detected on the liver surface (Fig. 5d). Histologically, hepatic adenomas and well-differentiated HCC were observed in Brg1^−/−^/c-MET and Brg1^−/−^/NRAS^V12^ mice (Fig. 5d). Tumor cells were proliferative, as demonstrated by Ki67 immunostaining (Fig. 5d). Importantly, immunolabeling with the Brg1 antibody revealed that HCC cells were derived from Brg1(−) hepatocytes and did not express Brg1 (Fig. 5d).

As overexpression of c-MET occurs frequently in human HCCs, whereas *NRAS* mutations are rather rare, we focused on the Brg1^−/−^/c-MET mouse HCC model for additional studies. At the biochemical level, ectopically injected c-MET and activation of p-Akt and p-Erk downstream of c-MET were detected in Brg1^−/−^/c-MET mouse HCC tissues (Supplementary Fig. 12). It is worth to note that tumor development was rather slow in these mice (Supplementary Fig. 13A). For instance, c-MET and activated mutant form of β-Catenin (β-CateninS45Y) are able to induce HCC formation within 8 weeks post injection^27^. Similarly, loss of Pten (sgPten) synergizes with c-MET to promote hepatocarcinogenesis within ~11–17 weeks post injection^28^. Furthermore, although c-MET/β-CateninS45Y mouse HCC tissues expressed high levels of alpha-fetoprotein (Afp), Brg1^−/−^/c-MET HCCs expressed Afp at very low levels, similar to those in normal liver (Supplementary Fig. 13B). We investigated the correlated between AFP and BRG1 in human HCC samples using the TCGA data set. We found that there is a statistically significant positive correlation between BRG1 expression and AFP expression in human HCCs (Supplementary Fig. 13C). Thus, Brg1^−/−^/c-MET mice could represent an excellent Afp(−) murine HCC model.

Altogether, our investigation demonstrates that Brg1 can function as a tumor suppressor, and loss of *Brg1* is able to cooperate with c-MET or NRAS^V12^ to induce liver tumor development in mice.

## Discussion

SWI/SNF complex is an important chromosome remodeling factor and regulates the expression of genes in the cells. BRG1 and BRM are two Helicases/ATPases in the SWI/SNF complex. Although BRG1 is part of the BAF complex, BRM is found in both BAF and PBAF complexes. Importantly, the presence of BRM and BRG1 is mutually exclusive in the BAF complex^29^. Mounting evidence indicates that the deregulation of SWI/SNF complex contributes to cancer development, including HCC. For instance, *ARID1A* and *ARID2* are among the most frequently mutated genes in HCC^30^. However, the functional roles of BRG1 in liver carcinogenesis remain controversial. It was found that a SNP in *SMARCA4* gene, namely rs11879293, is associated with decreased risk of developing HCC^31^. In human HCCs, loss-of-function mutations of *SMARCA4* are present in a small percentage of samples^30^, and the homozygous deletion of *SMARCA4* was detected in the SNU398 HCC cell line^32^. These results support a tumor suppressor role for BRG1 in HCC. However, multiple studies have demonstrated that BRG1 expression is upregulated in human HCCs. Overexpression of BRG1 in HCC cells promotes cell proliferation and invasion^19^. These results suggest that BRG1 may paradoxically function as an oncogene in HCC as well.

In this manuscript, we systematically analyzed BRG1 expression patterns and *SMARCA4* mutation status in human HCC samples. In addition, we investigated the functional roles of Brg1 in hepatocarcinogenesis using mouse modeling. Consistent with previous reports, we found that BRG1 expression is upregulated in most of human HCC samples (Fig. 1 and Supplementary Fig. 2). Importantly, we established a BRG1-correlated gene expression signature. Pathway analysis demonstrated that the high expression of BRG1 correlates with cell cycle and DNA replication, whereas low expression of BRG1 correlates with liver metabolism. These data clearly support the finding that high BRG1 expression is associated with increased cell proliferation and decreased hepatocyte differentiation in human HCCs. This observation is further supported by the fact that high BRG1 levels correlate with poor patient survival.

To further validate the oncogenic roles of BRG1, we applied mouse genetic approaches. We found that c-MYC-induced mouse HCC exhibit high levels of BRG1 expression. We analyzed BRG1-correlated genes in c-MYC mouse HCC samples. We found that genes correlated with high BRG1 are also enriched in c-MYC HCC upregulated gene list, whereas genes correlated with low BRG1 are enriched in c-MYC HCC downregulated gene list (Supplementary Fig. 10). These bioinformatics analyses provide unbiased support of the activation of BRG1 in c-MYC induced liver cancer development. Next, we expressed c-MYC while simultaneously deleting *Brg1* in mouse hepatocytes. We discovered that ablation of *Brg1* completely prevented c-MYC driven HCC formation in vivo. The study, for the first time, provides in vivo evidence supporting the oncogenic role of BRG1 along hepatocarcinogenesis. It is worth to note that using c-MYC transgenic mice, Sun et al.^33^ showed that loss of *Arid1a*, another member of the SWI/SNF family, hampered c-MYC-driven HCC initiation in mice. Altogether, our data indicate the key role of the SWI/SNF chromosomal remodeling factors in c-MYC-induced hepatocarcinogenesis. It is well-known that c-MYC is a major oncogene in HCC, whose direct targeting has proven to be difficult. Thus, our study suggests that targeting BRG1 or other members of the SWI/SNF complexes may be an alternative approach to target HCC with c-MYC amplification and/or overexpression.

Although BRG1 expression is found to be upregulated in the vast majority of human HCC samples, it is worth to note that a small percentage of HCCs have low expression of BRG1 or harbor inactivating *SMARCA4* mutations, suggesting a possible tumor suppressor role of BRG1. Importantly, in a previous study, BRG1 has been shown to possess both oncogenic and tumor suppressor roles in pancreatic cancer^34^. In addition, the study by Sun et al.^33^ demonstrated that ARID1A has context-dependent roles as tumor suppressor and oncogene in liver cancer. Therefore, we investigated the possible tumor-suppressing functions of Brg1 in mice and found that deletion of Brg1 cooperates with activated Ras/MAPK signaling (via either c-MET or NRAS^V12^ overexpression) to promote HCC development in mice. Comparing with other common oncogenic signals, such as loss of Pten or activation of the Wnt/β-Catenin, HCC induced by loss of Brg1 were characterized by longer tumor latency. Previously, we have established multiple HCC models by the combination of oncogenes, such as Akt/NRas(V12)^35^, sgPten/c-Met^28^, c-Met/β-Catenin^36^, and c-Met/sgAxin1^37^. All these HCC models have high levels of Afp. Interestingly, Brg1^−/−^/c-MET mouse HCCs are Afp(−). Consistently, we have found that low expression of BRG1 is associated with low AFP levels in human HCC samples. To the best of our knowledge, Brg1^−/−^/c-MET HCC is the first murine HCC model with low Afp. This model could be very useful in studying therapeutic approaches against Afp(+) and Afp(−) HCC.

One of the key conclusion from our study is that BRG1 has context-dependent tumor-suppressing or oncogenic roles during hepatocarcinogenesis. It is worth to note that there is increasing evidence, highlighting the importance of context-dependent control of gene expression in cancer. The molecular mechanisms underlying the dual role of BRG1 remain unclear. Shi et al.^38^ discovered that BRG1 functions as an oncogene in leukemia via regulating c-MYC and its transcriptional program. In human HCC samples, SMARCA4 and MYC are found to be in a gene-regulatory network^39^. The results are consistent with our current study indicating that Brg1 is required for c-MYC-driven HCC formation. The biochemical cross-talk between BRG1 and c-MET has not been reported previously. Our investigation, therefore, provides novel insights into how these two pathways may act in concert to induce HCC development. Obviously, further experiments are required to elucidate the mechanisms by which BRG1 functions to regulate hepatocarcinogenesis.

At present, precision medicine has become a norm in oncology. It is important to recognize that there is no one-size-fits-all therapy. Although some mutations or genetic alternations are relatively rare, they may represent excellent opportunities for targeted therapy. Low BRG1 expression or loss of function mutations of *SMARCA4* may only account for a total of ~5% of HCC. However, these HCCs may be sensitive to specific treatment strategies. For instances, previous studies have shown that EZH2 inhibition sensitizes *SMARCA4* mutant lung cancers to Topoisomerase 2 inhibitors^40^. BRG1-deficient cancer was found to be highly sensitive to BRM/SMARCA2 inhibition^41^. It would be very useful to test these treatment options in BRG1 low or *SMARCA4* mutant HCCs, and Brg1^−/−^/c-MET mouse HCC represents an excellent murine HCC model to challenge these treatment strategies.

In summary, BRG1 possesses oncogenic and tumor suppressive roles that are context and gene dependent. A better understanding of the consequences of either overexpression or loss of BRG1 in HCC would be necessary for the establishment of effective treatments against this deadly disease.

## Materials and methods

### Human data retrieval and analysis

The data illustrated here were generated by the Cancer Genome Atlas (TCGA) Research Network: https://www.cancer.gov/tcga and the Catalogue of Somatic Mutations in Cancer (COSMIC): https://cancer.sanger.ac.uk/cosmic. We used TCGA expression data that were frozen in 2/25/2015. The overall sample size is 410, including 50 surrounding liver tissues (ST) and 360 primary hepatocarcinoma (HCC) from TCGA LIHC database. The mutation and copy number alteration data were extracted with cBioportal (http://www.cbioportal.org)^42,43^. Survival plot was obtained from UALCAN^44^. Data were analyzed and visualized in R using multiple packages. Heatmap was drawn with Complexheatmap^45^. The BRG1 expression data were normalized with Mean plus 1.5-fold SD of the ST group and ordered ascendingly. Mutations on TP53, CTNNB1, AXIN1, TSC1, TSC2, ARID1A, ARID2, and BRG1 were also included in the heatmap. Scatter-bar plot of the mRNA expression of TCGA and Fudan data sets were drawn with Graphpad Prism 7.0 (GraphPad Software, San Diego, US). Scatter plot of correlation was made using ggplot2 (*H. Wickham. ggplot2: Elegant Graphics for Data Analysis. Springer-Verlag New York, 2016*.) and DEGreport (*Pantano L 2014; DEGreport: Report of DEG analysis. R package version 1.0.0*.). Pearson correlation was used to test correlation between the log2 mRNA expression of different genes with BRG1. Positively co-expressed genes and negatively co-expressed genes were extracted from cBioportal with a threshold of |*r*| > 0.3 and *p* value < 0.05 (Supplementary Table 4). Subsequently, we conducted KEGG and GO pathway analysis using limma^46^, GO.db (*Carlson M 2019; GO.db: A set of annotation maps describing the entire Gene Ontology. R package version 3.8.2*.) and KEGGREST (*Tenenbaum D 2019; KEGGREST: Client-side REST access to KEGG. R package version 1.24.0*.). Top 20 KEGG pathways enriched in BRG1 positively correlated or negatively correlated genes were visualized by scatter plot. Top 30 GO (Biological Process) pathways were visualized by bar plot.

### Human tissue samples

A collection of 60 frozen HCC and corresponding non-tumorous surrounding livers was used. Tumors were divided in HCC with shorter/poorer (HCCP; *n* = 29) and longer/better (HCCB; *n* = 31) survival, characterized by < 3 and ≥ 3 years’ survival following partial liver resection, respectively. The clinicopathological features of liver cancer patients are summarized in Supplementary Table 5 HCC specimens were collected at the Medical Universities of Regensburg (Regensburg, Germany) and Sassari (Sassari, Italy). Institutional Review Board approval was obtained at the local Ethical Committee of the Medical Universities of Regensburg and Sassari. Informed consent was obtained from all individuals.

### RNASeq analysis of mouse liver tissues

RNA was extracted from AAV-Cre and AAV-Null infected mouse liver tissues. RNAseq libraries were generated as previously described^47^. In brief, total RNAs were reverse transcribed by SuperScript II (Invitrogen, Cat#18064-014) with oligo-dT and LNA-containing TSO primers. cDNAs were pre-amplified by using KAPA HIFI HotStart ReadyMix (Kapabiosystems Cat#KK2602) with IS PCR. PCR amplified cDNAs were purified by Ampure XP beads (Beckman Coulter, Cat#A63881) and sonicated to 200–400 bps by Bioruptor Pico (Diagenode). Sonicated cDNAs were used for RNAseq library preparation and sequencing as described previously^47^.

### Plasmids and reagents

The plasmids used for the study, including pT3EF1α-c-MYC, pT3EF1α-c-MET, pT2-Caggs-NRAS^V12^, pCMV, pCMV-Cre, and pCMV/sleeping beauty transposase (SB) have been described in our previous publications^48,49^. All plasmids were purified by using the Endotoxin free Maxi prep kit (Sigma-Aldrich, St. Louis, MO) before injection. The AAV8.TBG.PI.Cre.rBG and AAV8.TBG.PI.Null.Bgh vectors were obtained from the University of Pennsylvania Gene Therapy Core Services.

### Animals and treatments

*Brg1*^*flf*^ mice (C57BL/6 background) were kindly provided to us by Dr. Matthias Hebrok of UCSF^50^. Mice were crossed for six generations with FVB/N mice to generate *Brg1*^*flf*^ mice in FVB/N background. To deplete Brg1 gene specifically in adult mouse hepatocytes, six-week old *Brg1*^*flf*^ mice were infected with 4 × 10^11^ genome copies adeno-associated viral with TBG promoter-Cre recombinase (AAV8.TBG.PI.Cre.rBG) by tail vein injection and waited 3 weeks for gene deletion. An AAV8.TBG.PI.Null.Bgh was injected into additional *Brg1*^*flf*^ mice and used as control. Sleeping beauty-mediated hydrodynamic injection was performed as described^24^. The detailed plasmid mixture information is reported in Supplementary Table 6. Mice were housed, fed, and monitored in accord with protocols approved by the Committee for Animal Research at the University of California San Francisco (San Francisco, CA). Mice were closely monitored for liver tumor development as palpable abdominal masses. Mice were killed at indicated time points or when they became moribund or develop large abdominal masses. Liver weight, body weight, as well as surface tumor nodule numbers were recorded.

### Immunohistochemistry (IHC)

Liver specimens were fixed in 4% paraformaldehyde overnight at 4 °C then embedded in paraffin. Tissue sections were cut from paraffin blocks at 5 μm in thickness. For immunohistochemical staining, the slides were deparaffinized and then microwaved in 10 mmol/L citrate buffer (pH 6.0) for 10 minutes for antigen retrieval. After a 20 minutes cool down at room temperature, the slides were blocked using 5% goat serum and Avidin-Biotin blocking kit (Vector Laboratories, Burlingame, CA). Next, the slides were incubated with primary antibodies overnight at 4 °C. The following primary antibodies were used: anti-Ki-67 (MA5-14520, Thermo Fisher Scientific) and anti-Brg1 (110641, Abcom). The immunoreactivity was visualized with the Vectastain Elite ABC kit (Vector Laboratories, Burlingame, CA), using Vector NovaRED (Vector Laboratories) as the chromogen. Slides were then counterstained with hematoxylin solution (ThermoFisher Scientific, Pittsburg, PA).

### Protein extraction and western blot analysis

Mouse liver tissues and cells were homogenized in M-PER Mammalian Protein Extraction Reagent (ThermoFisher Scientific) containing the Halt Protease Inhibitor Cocktail (ThermoFisher Scientific). Subsequently, protein concentrations were determined by using the Pierce Microplate BCA Protein Assay Kit (ThermoFisher Scientific). For western blotting, proteins were boiled in Tris-Glycine SDS Sample Buffer (Bio-Rad) for denaturation, separated by sodium dodecyl sulfate polyacrylamide gel electrophoresis, and transferred onto nitrocellulose membranes (Bio-Rad). Membranes were blocked in 10% non-fat milk in Tris-buffered saline containing 0.05% Tween-20 for 1 hour at room temperature and then incubated with primary antibodies at 4 °C overnight. Afterwards, membranes were incubated with a horseradish peroxidase-secondary antibody (1:5000; Jackson ImmunoResearch Laboratories Inc., West Grove, PA) at room temperature for 1 hour. After appropriate washing, membranes were developed with the Super Signal West Dura Kit (ThermoFisher Scientific, Waltham, MA). The following antibodies were used: anti-Brg1 (110641, Abcam), anti-c-MET (71-8000, Invitrogen), anti-total AKT (9272, Cell Signaling Technology), anti-Phospho-AKT (Ser473; 3787, Cell Signaling Technology), anti-Phospho-AKT (Ser308; 13038, Cell Signaling Technology), anti-Phospho-ERK1/2 (4370, Cell Signaling Technology), anti-total ERK1/2 (9102, Cell Signaling Technology), anti-GAPDH (5174, Cell Signaling Technology).

### Quantitative reverse transcription real-time polymerase chain reaction (qRT-PCR)

Total RNA was extracted from liver tissues or cells using the Quick-RNA MiniPrep Kit (Genesee Scientific, El Cajon, CA). cDNA was generated using the 5 × iScriptTM RT Supermix (Bio-Rad), according to the instructions of the manufacturer. The real-time quantitative RT-PCR (RT-qPCR) was performed using the TaqMan Universal PCR Master Mix (Thermo Fisher Scientific) on an ABI Prism 7000 Sequence Detection System (Applied Biosystems, Foster City, CA). Analysis of mRNA levels was conducted with the QuantStudio Real-Time PCR software version 1.1 (Thermo Fisher Scientific). The RT-qPCR was conducted as follows: template denaturation at 95 °C for 10 minutes, primer annealing at 95 °C for 15 s, and extension step at 60 °C for 1 minute. Forty cycles of amplification were used. All the primers used in the present study are listed in Supplementary Table 7. To determine the levels of *BRG1* in our HCC collection, Gene Expression Assays for human *BRG1/SMARCA4* (ID# Hs00231324_m1) and β-Actin (ID #4333762 T) genes were purchased from Applied Biosystems (Foster City, CA, USA). Quantitative values for each were calculated by using the PE Biosystems Analysis software and expressed as number target (NT). *NT* = 2^−ΔCt^, wherein ΔCt value of each sample was calculated by subtracting the average Ct value of the target gene from the average Ct value of the *β-Actin* gene.

### Statistical analysis

The investigators were blinded to the group allocation during the experiment. Sample size for each experiment was determined by power analysis (power of 0.8 with alpha value 0.05). Animals were randomly allocated to experimental groups and processed. No animals were excluded from the analyses. All experiments were performed at least three times. All data are presented as means ± SD for each group. Statistical differences between two groups were determined using the *U* tests embedded in the Prism 6 software version 6.0 (Graph Pad Software Inc., La Jolla, CA). *P* < 0.05 was considered statistically significant. Our data follow a normal distribution, and the variances among the groups are similar.

## Supplementary information

Supplementary Figure Legends

Supplementary Table Legends

Supplementary Figure 1

Supplementary Figure 2

Supplementary Figure 3

Supplementary Figure 4

Supplementary Figure 5

Supplementary Figure 6

Supplementary Figure 7

Supplementary Figure 8

Supplementary Figure 9

Supplementary Figure 10

Supplementary Figure 11

Supplementary Figure 12

Supplementary Figure 13

Supplementary Table
