# Supplementary material for: Oncogene-dependent function of BRG1 in hepatocarcinogenesis
Source: Cell Death Dis. 2020 Feb 4;11(2):91. doi: 10.1038/s41419-020-2289-3 (PMC7000409; doi:10.1038/s41419-020-2289-3)
Supplement: Supplementary file 1 — Supplementary Figure Legends [file 41419_2020_2289_MOESM1_ESM.docx]

**Supplementary Figure Legends**

**Supplementary Fig. 1 BRG1 gene is overexpressed and associated with an adverse outcome in human hepatocellular carcinoma (HCC) patients.** (A) Levels of BRG1 mRNA were significantly more elevated in HCC (n = 60) than corresponding non‐tumorous surrounding livers (ST; n = 60) and normal livers (NL; n=5), as detected by quantitative reverse‐transcription PCR. Number target (NT) = 2^−ΔCt^, wherein ΔCt value of each sample was calculated by subtracting the average Ct value of the BRG1 gene from the average Ct value of the β‐actin gene. Mann–Whitney test: **, *P* < 0.001 vs. NL; ***, *P* < 0.0001 vs. ST. (B) Levels of BRG1 mRNA were significantly higher in HCC with poorer outcome (HCCP) than in tumors with better prognosis (HCCB). Student‘s t test: ***, *P* < 0.0001 vs. HCCB.

**Supplementary Fig. 2 Immunhistochemical (IHC) expression of BRG1 in humun hepatocellular carcinoma and surrounding hepatocyctes.** A‐C Hepatocellular carcinoma with a high (A), intermediate (B), and low (C) expression of BRG1; (D) Minimal expression in the surrounding hepatocyctes, note only a strong expression in a biliary duct, lymphocytes and macrophages; (E)IHC scores of 16 paired surrounding liver tissue (ST) and HCC (T). Data are presented as mean±SD. * *P*<0.05, ** *P*<0.01, *** *P*<0.001 when compared to ST.

**Supplementary Fig. 3 Mutations on BRG1 in TCGA (A) and COSMIC (B) human HCC databases.**

**Supplementary Fig. 4 Correlation scatterplot of BRG1 with CDK4, CCNE1 and CCNB1 in TCGA HCC dataset. Pearson correlation was applied in calculations.**

**Supplementary Fig. 5 Gene Ontology analysis of BRG1 positively (A) and negatively (B) coexpressed genes in TCGA HCC dataset.**

**Supplementary Fig. 6 Differentially expressed analysis between AAV‐Cre and AAV‐Null mouse liver tissue.** Volcano plot was drawn using ‐log10 (adjusted p‐value) vs log2 (fold change). Differentially expressed genes are shown in red dots with the most DE genes labeled.

**Supplementary Fig. 7 Compared gene expression patterns of liver tissues from AAV‐Null and AAV‐Cre mice using RNASeq.**

**Supplementary Fig. 8 Relative expression of differentially expressed gene identified via RNASeq using qRT‐PCR.** Data are presented as mean±SD. * *P*<0.05, ** *P*<0.01, *** *P*<0.001 when compared to Brg1^f/f^ AAV‐Null.

**Supplementary Fig. 9 Brg1 is upregulated in c‐MYC HCC. Abbreviation: NT, no tumor; T, tumor.**

**Supplementary Fig. 10 Expression of Brg1 correlated genes in c‐MYC m use HCC samples.** (A, B) Heatmap analysis of Brg1 positively and negatively correlated genes in c‐MYC HCC; (C) % enrichment analysis of Brg1 positively and negatively correlated genes in c‐MYC HCC. Abbreviation: WT, normal liver from wild type mice.

**Supplementary Fig. 11 Deletion of Brg1 alone is unable to promote liver tumor.** (A)Study desigh; (B) Liver weight and liver body ration of Brg1f/f AAV‐Cre mice; (C) Gross image, H&E staining, Brg1 and Ki67 staining of Brg1^f/f^ AAV‐Cre mouse liver.

**Supplementary Fig. 12 Representative western blot analysis of p‐AKT and p‐ERK genes in Brg1^f/f^ AAV‐Null, Brg1^f/f^ AAV-Cre, c‐MET, and Brg1^‐/‐^/c‐MET Tumor tissues.**

**Supplementary Fig. 13 Characterization of Brg1^‐/‐^/c‐MET induce liver tumor.** (A) Comparison of Tumor incident rates of Brg1^‐/‐^/c‐MET mice, c‐MET/β‐Catenin mice and sgPten/c‐MET mice; (B) Afp mRNA expression in Brg1^‐/‐^/c‐MET and c‐MET/β‐Catenin mouse HCC tissues; (C) correlated between AFP and BRG1 in human HCC samples using the TCGA human HCC dataset. Data are presented as mean±SD. * *P*<0.05, ** *P*<0.01, *** *P*<0.001 when compared to FVB. Abbreviation: n.s, not significant.
