# Supplementary material for: Oncogene-dependent function of BRG1 in hepatocarcinogenesis
Source: Cell Death Dis. 2020 Feb 4;11(2):91. doi: 10.1038/s41419-020-2289-3 (PMC7000409; doi:10.1038/s41419-020-2289-3)
Supplement: Supplementary file 2 — Supplementary Table Legends [file 41419_2020_2289_MOESM2_ESM.docx]

**Supplementary Table Legends**

**Supplementary Table 1 BRG1 positively correlated genes in human HCCs using TCGA dataset.**

**Supplementary Table 2 BRG1 negatively correlated genes in human HCCs using TCGA dataset.**

**Supplementary Table 3 Raw data for the RNA analyses.**

**Supplementary Table 4 Positively co-expressed genes and negatively co-expressed genes were extracted from cBioportal.**

**Supplementary Table 5 Clinicopathological features of HCC patients.**

**Supplementary Table 6 Summary of plasmid mixture information.**

**Supplementary Table 7 Summary of primers.**
