# Supplementary figures and images for: Oncogene-dependent function of BRG1 in hepatocarcinogenesis
Source: Cell Death Dis. 2020 Feb 4;11(2):91. doi: 10.1038/s41419-020-2289-3 (PMC7000409; doi:10.1038/s41419-020-2289-3)

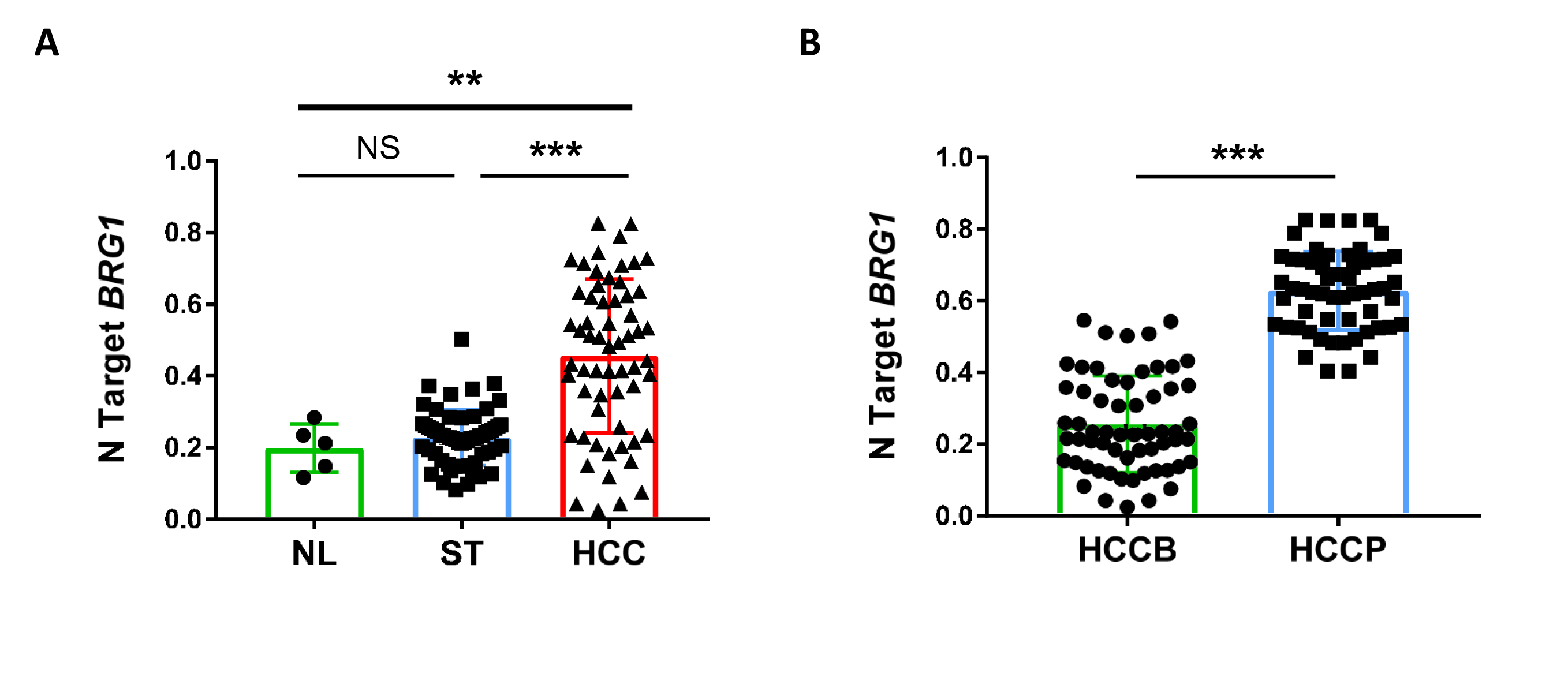

Supplement: Supplementary file 3 — Supplementary Figure 1 [file 41419_2020_2289_MOESM3_ESM.tif]

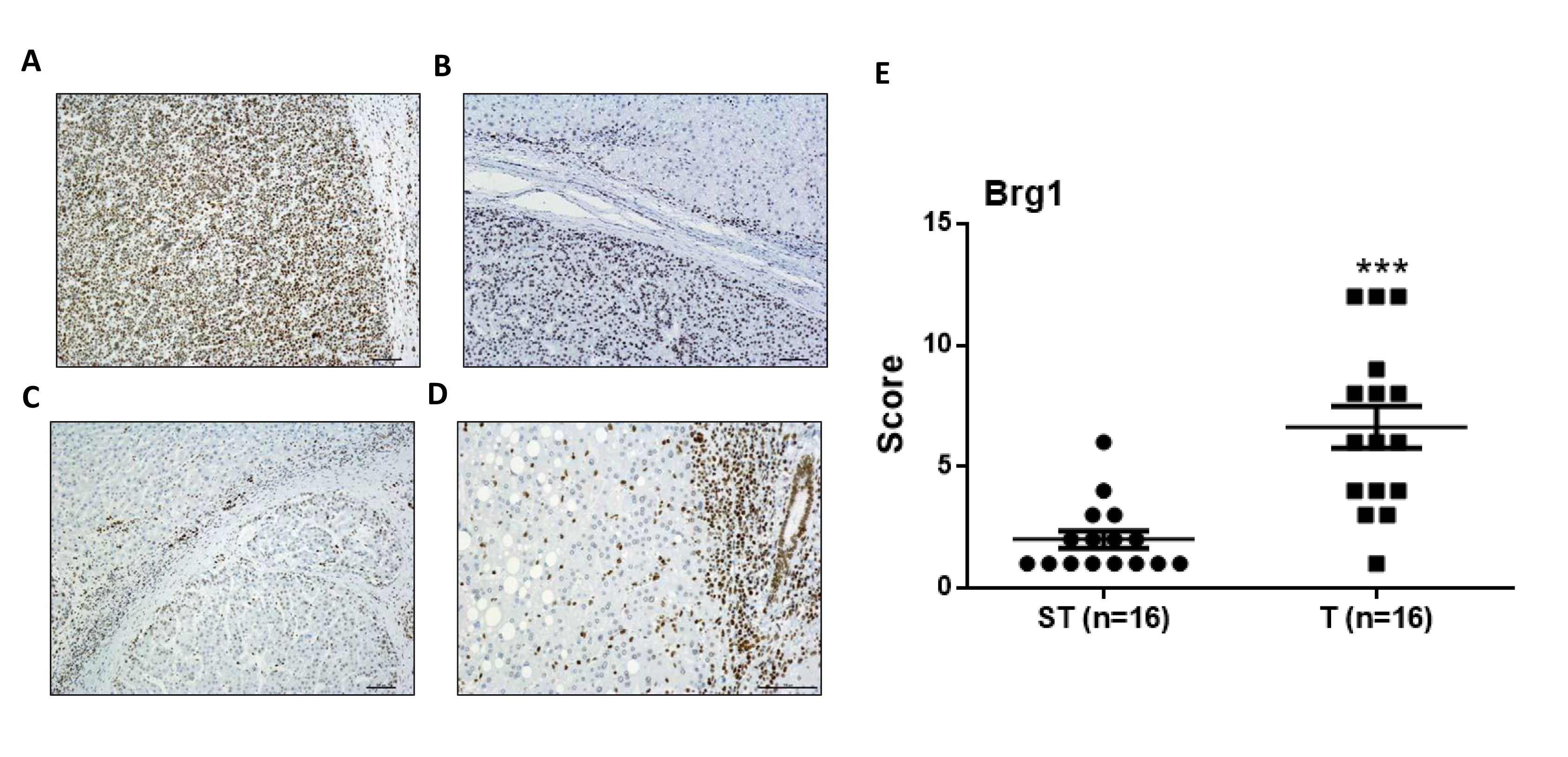

Supplement: Supplementary file 4 — Supplementary Figure 2 [file 41419_2020_2289_MOESM4_ESM.tif]

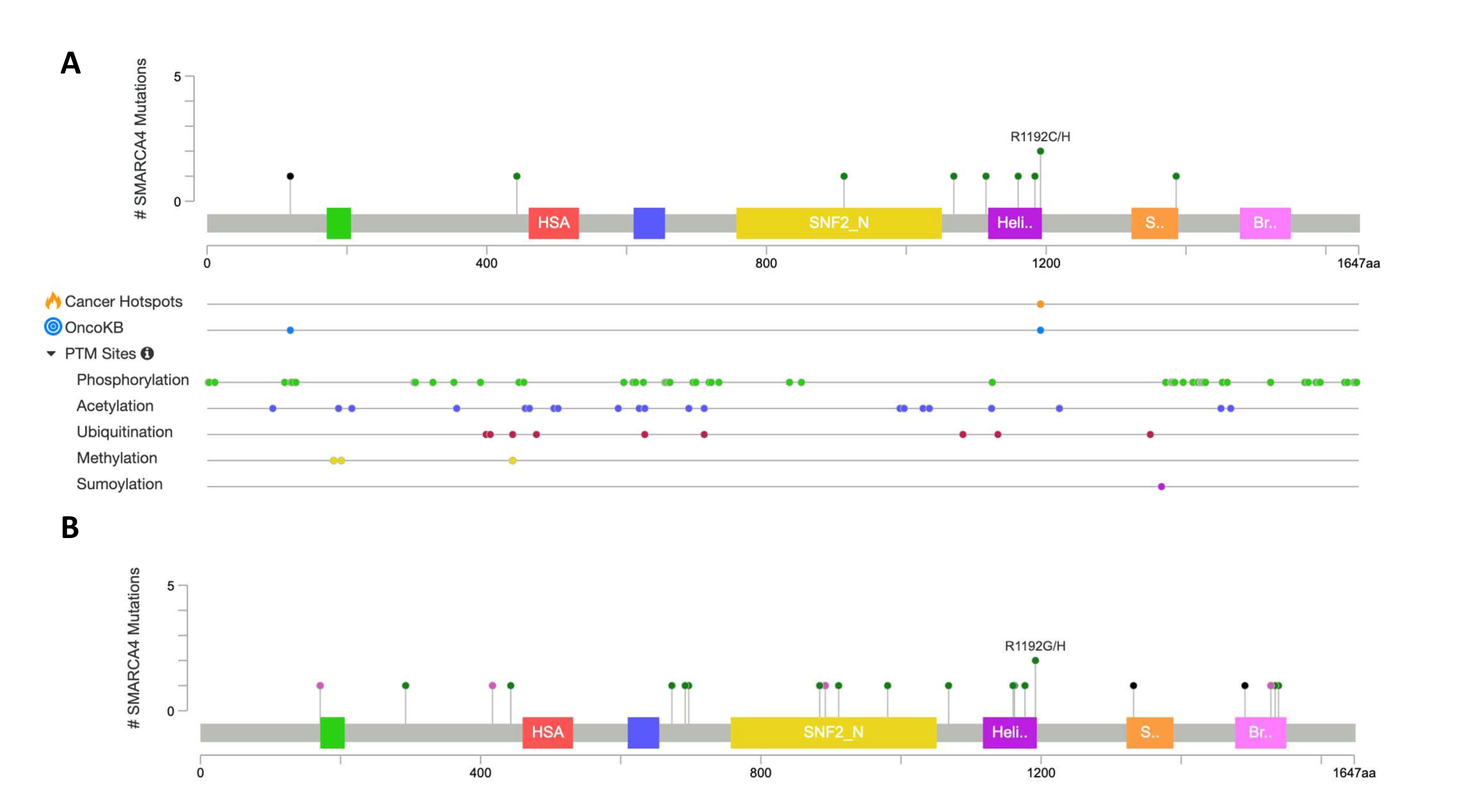

Supplement: Supplementary file 5 — Supplementary Figure 3 [file 41419_2020_2289_MOESM5_ESM.tif]

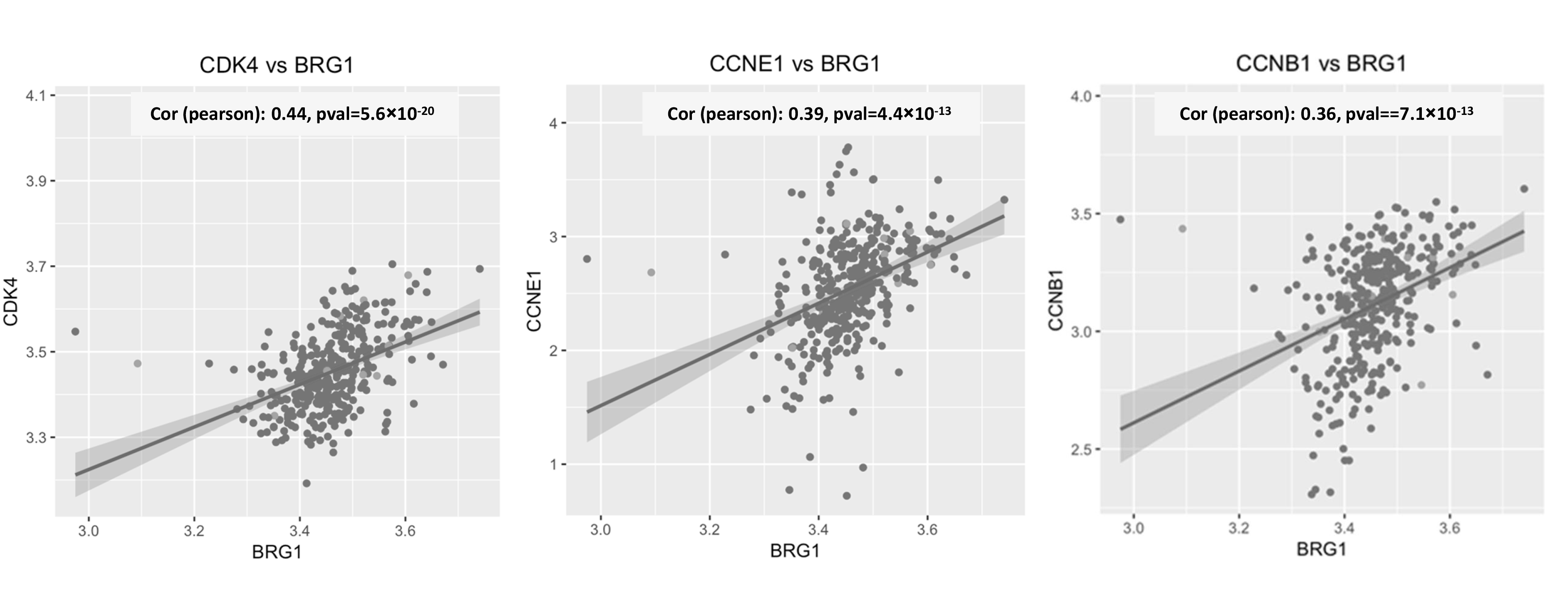

Supplement: Supplementary file 6 — Supplementary Figure 4 [file 41419_2020_2289_MOESM6_ESM.tif]

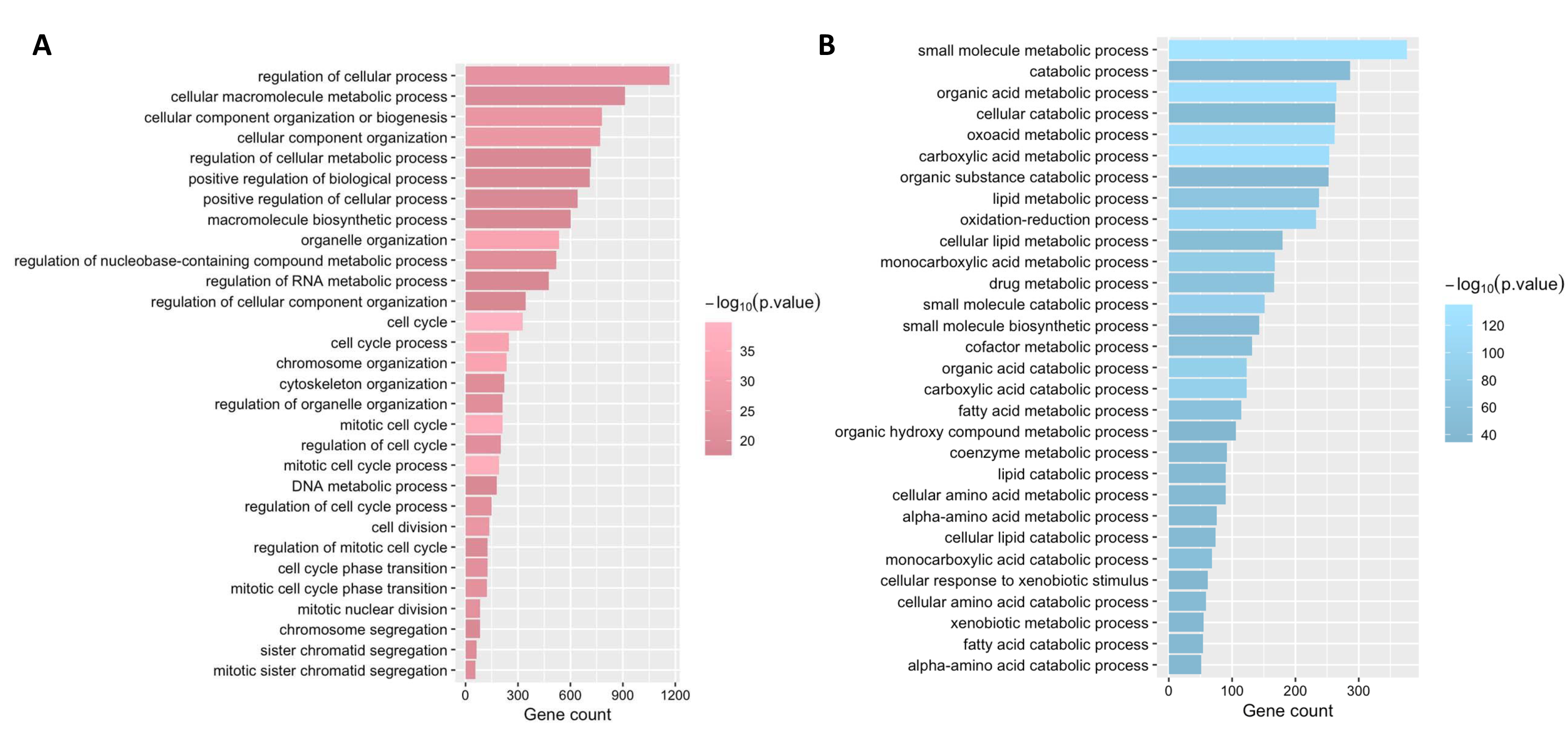

Supplement: Supplementary file 7 — Supplementary Figure 5 [file 41419_2020_2289_MOESM7_ESM.tif]

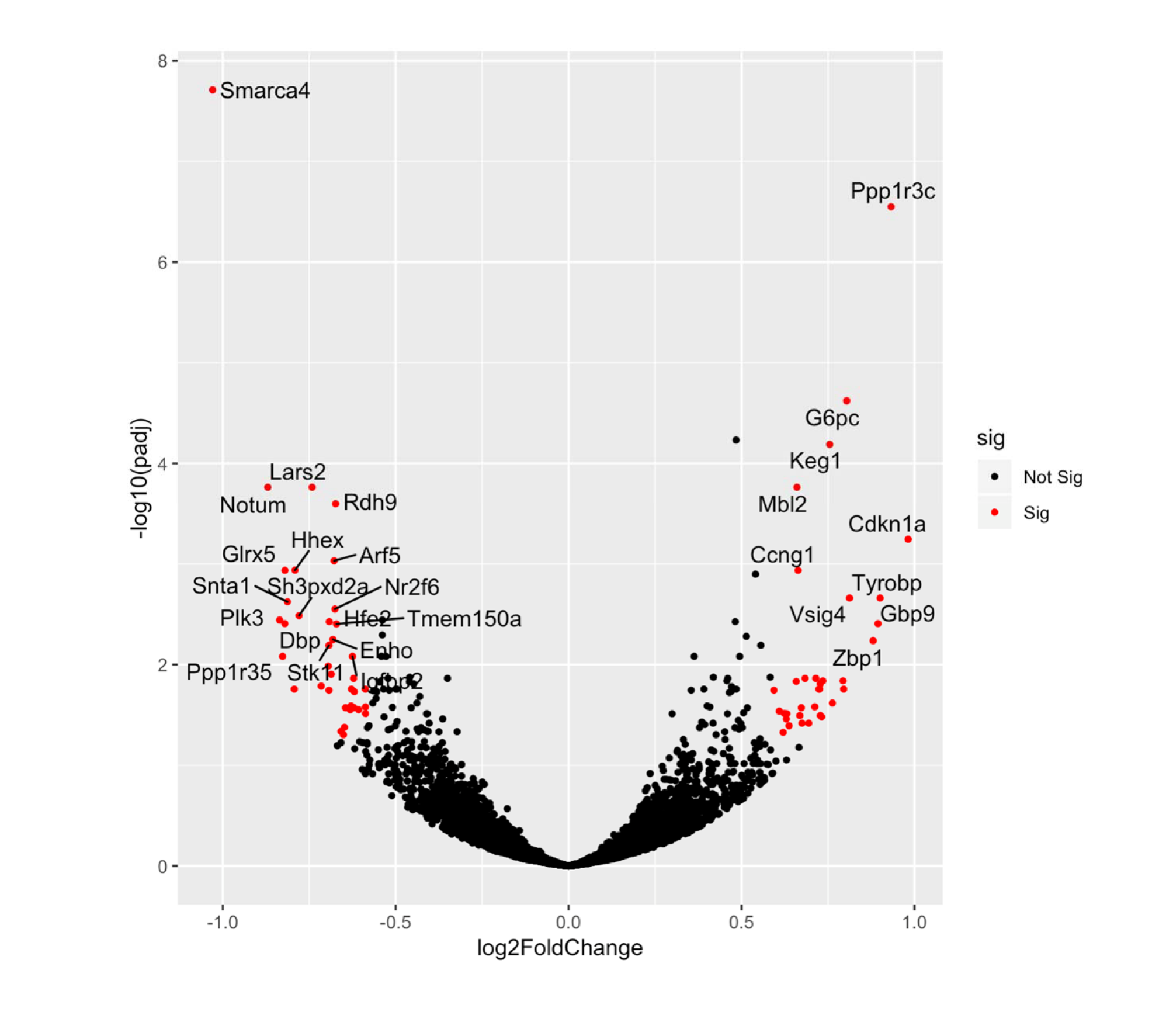

Supplement: Supplementary file 8 — Supplementary Figure 6 [file 41419_2020_2289_MOESM8_ESM.tif]

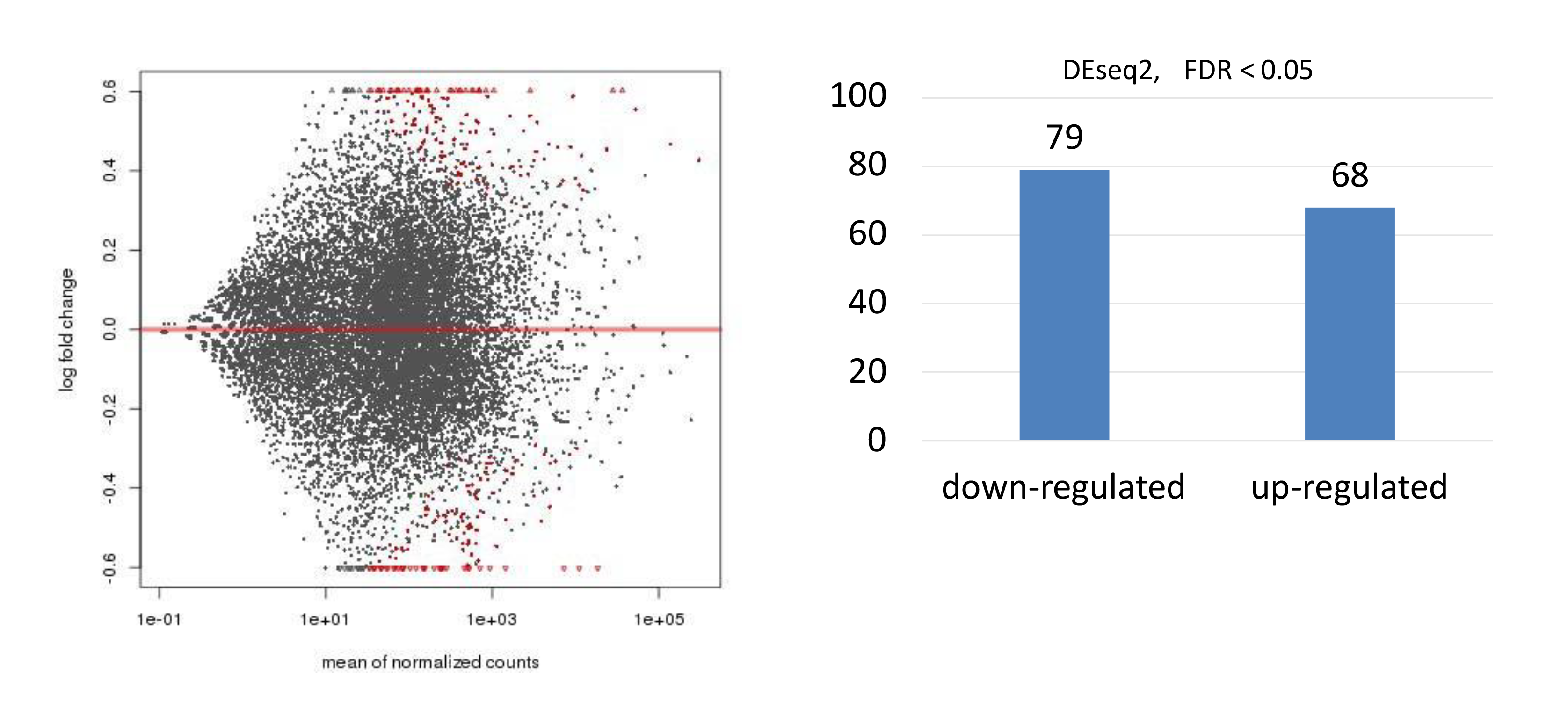

Supplement: Supplementary file 9 — Supplementary Figure 7 [file 41419_2020_2289_MOESM9_ESM.tif]

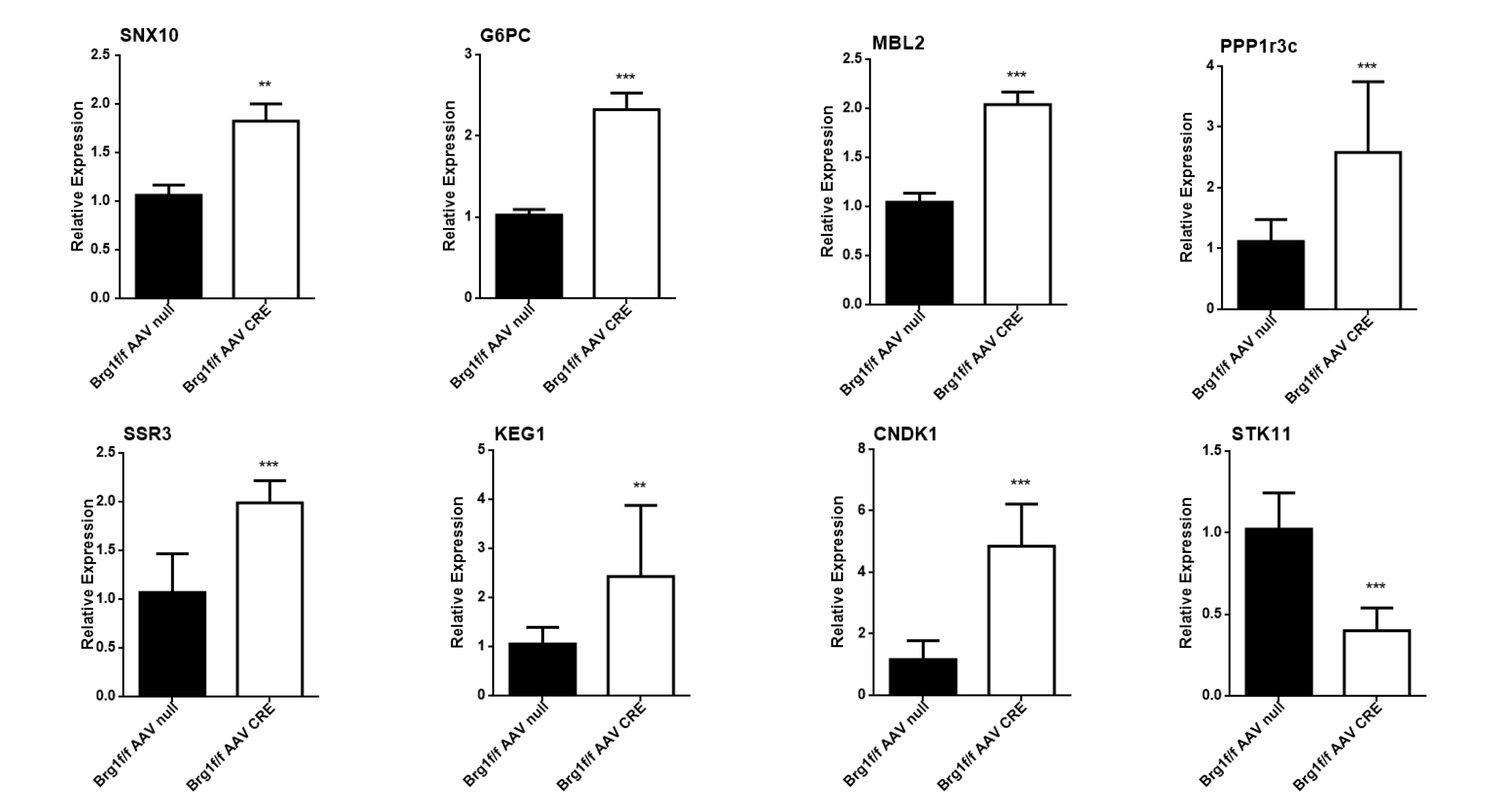

Supplement: Supplementary file 10 — Supplementary Figure 8 [file 41419_2020_2289_MOESM10_ESM.tif]

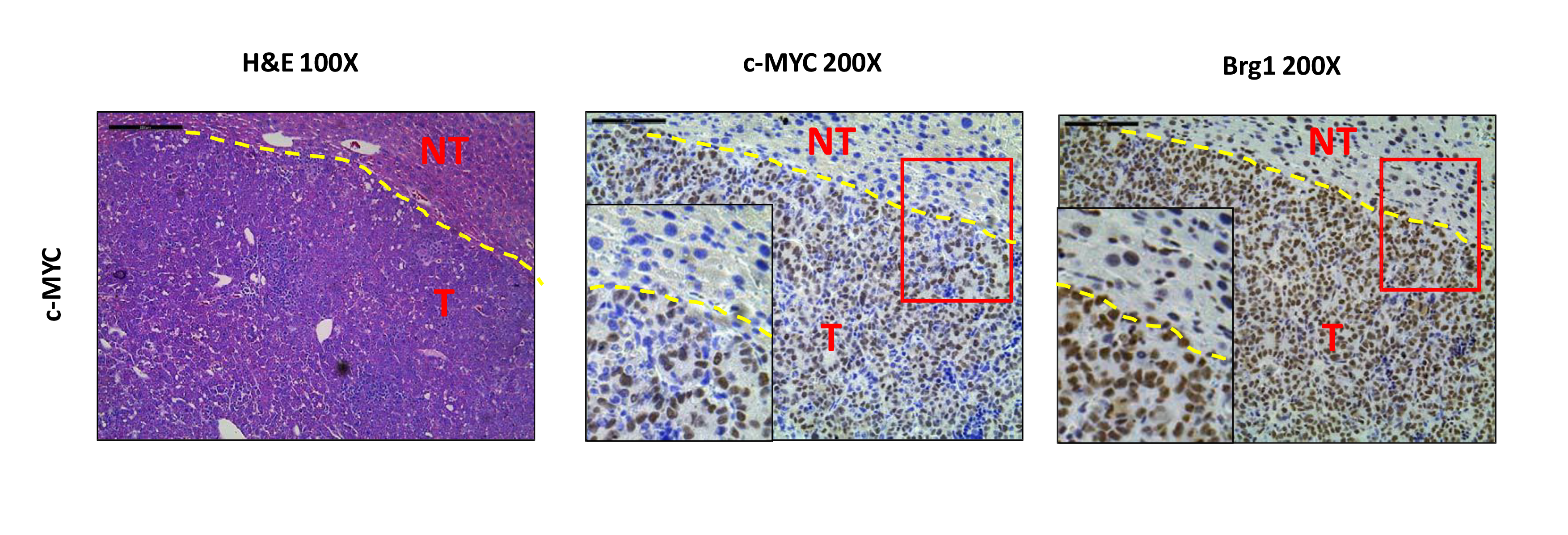

Supplement: Supplementary file 11 — Supplementary Figure 9 [file 41419_2020_2289_MOESM11_ESM.tif]

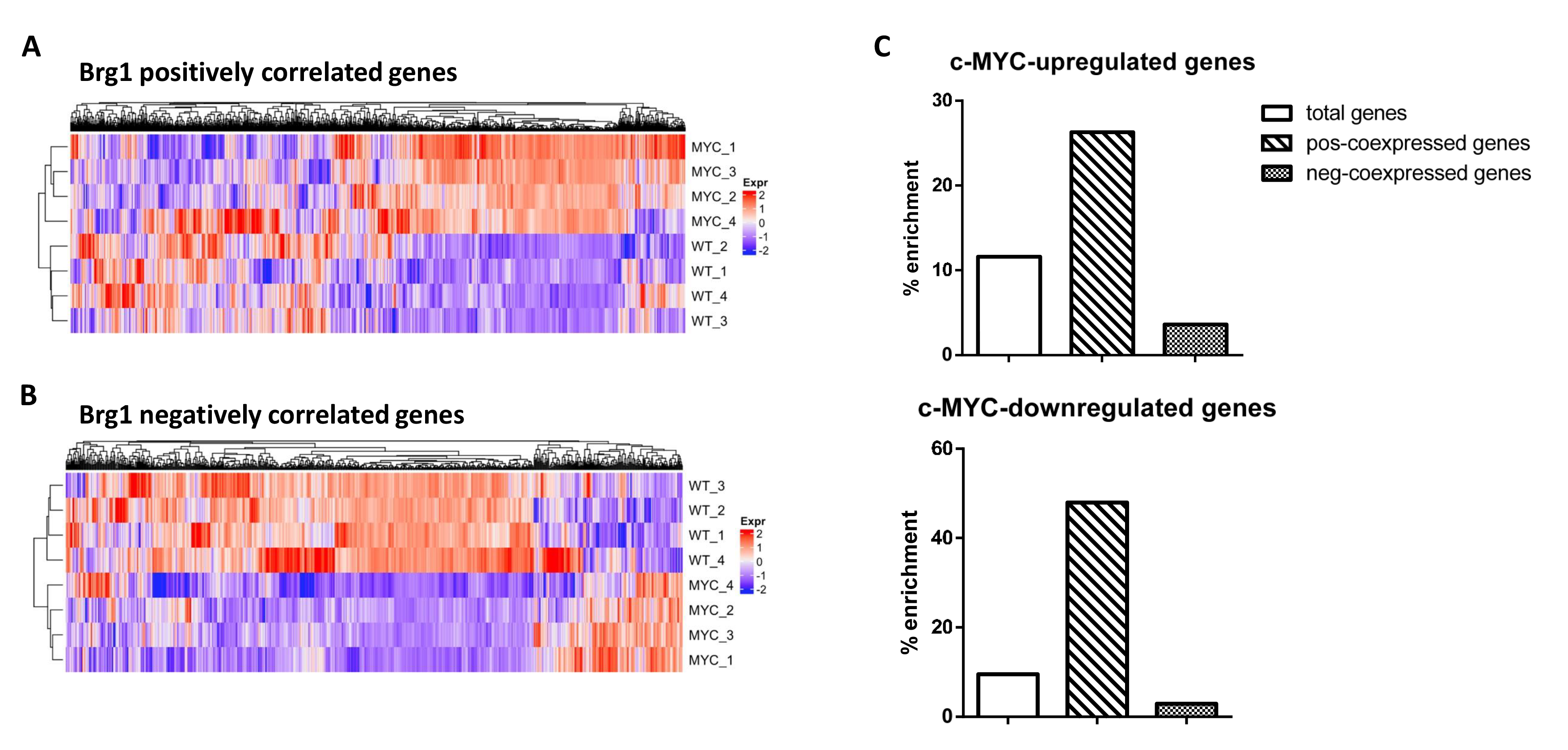

Supplement: Supplementary file 12 — Supplementary Figure 10 [file 41419_2020_2289_MOESM12_ESM.tif]

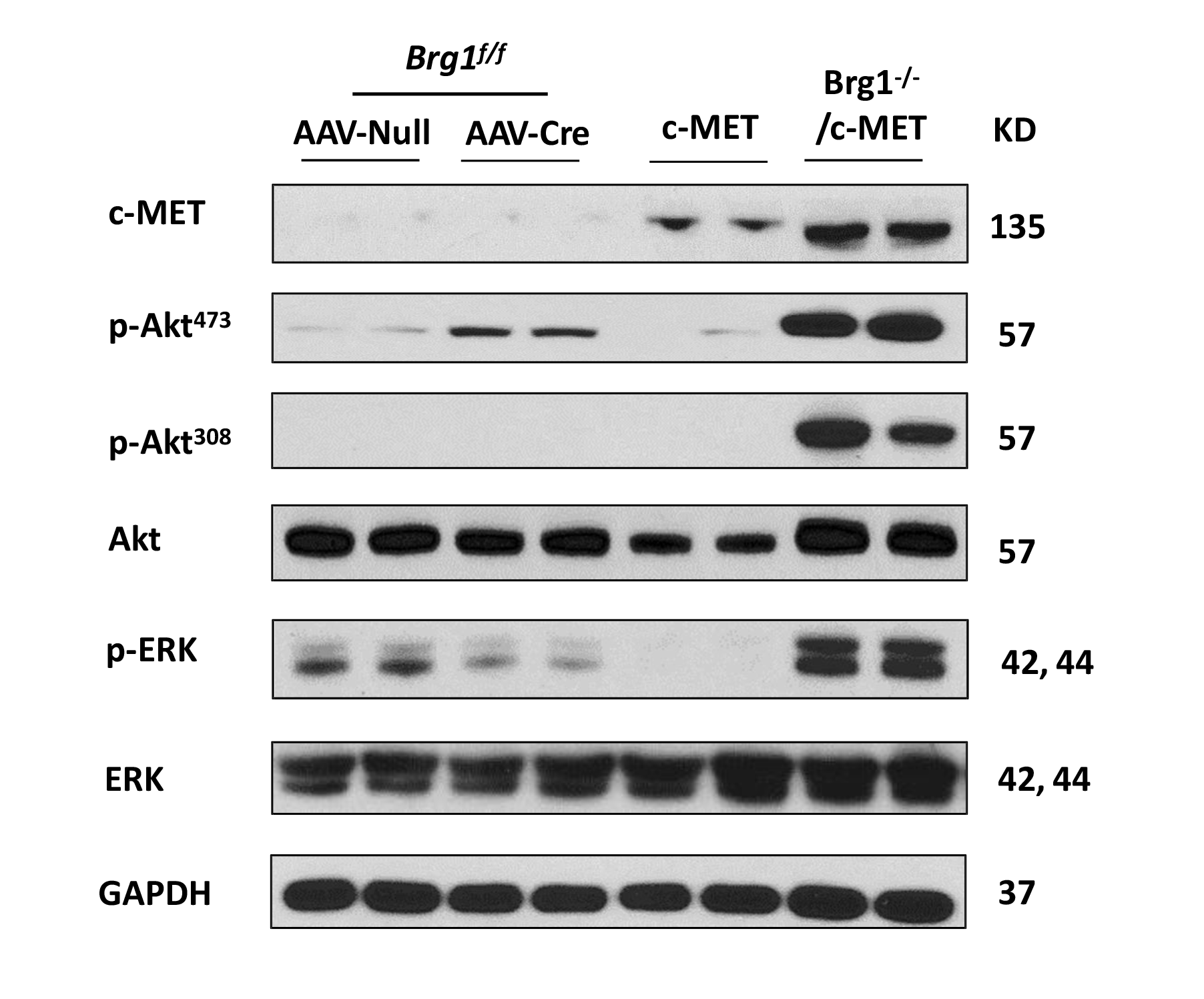

Supplement: Supplementary file 14 — Supplementary Figure 12 [file 41419_2020_2289_MOESM14_ESM.tif]

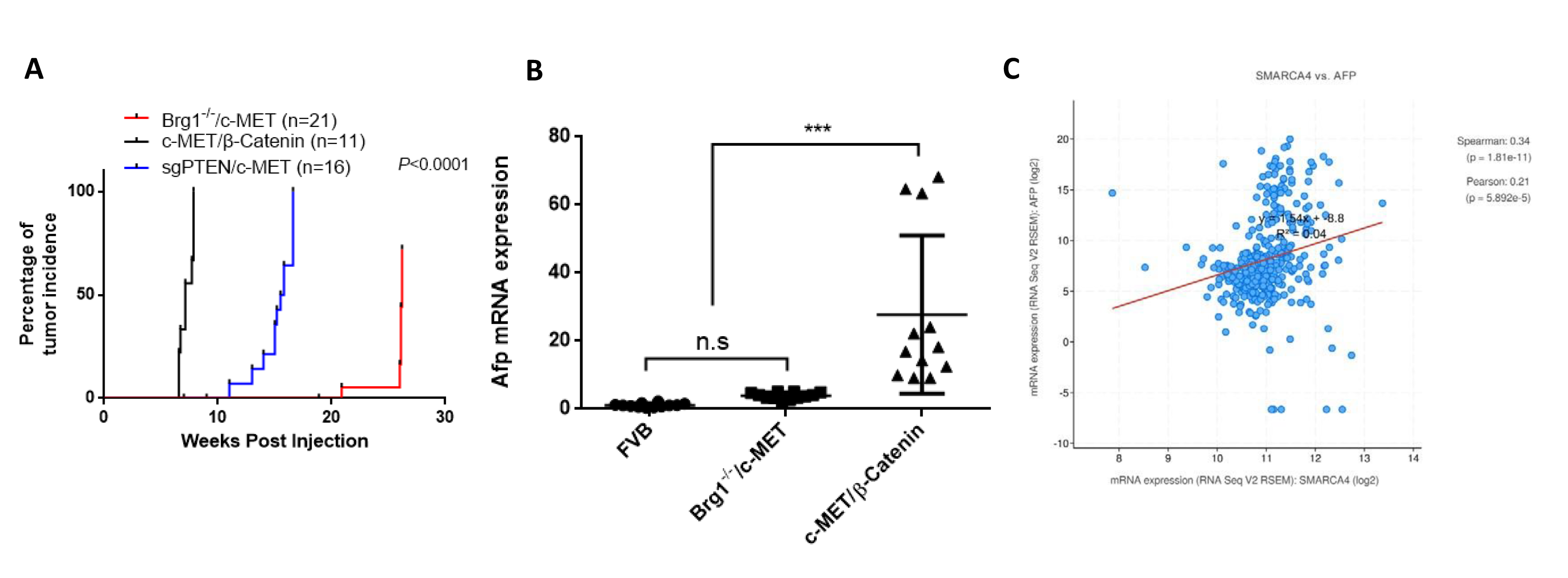

Supplement: Supplementary file 15 — Supplementary Figure 13 [file 41419_2020_2289_MOESM15_ESM.tif]
