# Supplementary material for: Oncogene-dependent function of BRG1 in hepatocarcinogenesis
Source: Cell Death Dis. 2020 Feb 4;11(2):91. doi: 10.1038/s41419-020-2289-3 (PMC7000409; doi:10.1038/s41419-020-2289-3)
Supplement: Supplementary file 13 — Supplementary Figure 11 [file 41419_2020_2289_MOESM13_ESM.pdf]

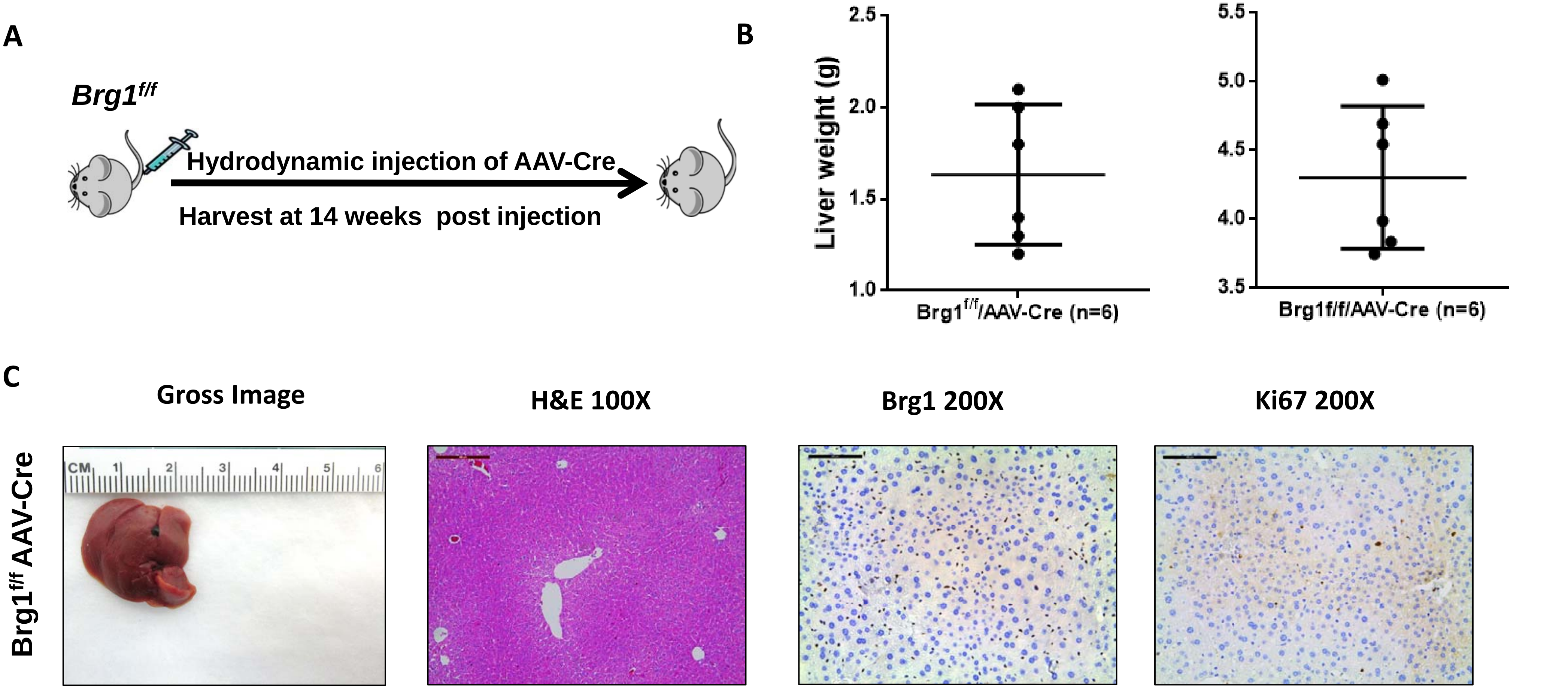

Supplementary Fig. 11 Deletion of Brg1 alone is unable to promote liver tumor. (A)Study desigh; (B) Liver weight and liver body ration of Brg1$^{f/f}$ AAV-Cre mice; (C) Gross image, H&E staining, Brg1 and Ki67 staining of Brg1$^{f/f}$ AAV-Cre mouse liver.
